# Mechanistic Model for Simulating Pesticide Uptake into Maize Pollen

**DOI:** 10.1021/acs.jafc.6c02800

**Published:** 2026-05-28

**Authors:** Arno Rein, Stefan Trapp, Klaus Hammel, Peter Fantke

**Affiliations:** † Chair of Hydrogeology, TUM School of Engineering and Design, 5205 Technical University of Munich, Arcisstr. 21, D-80333 Munich, Germany; ‡ Department of Environmental and Resource Engineering, Technical University of Denmark, Bygningstorvet 115, DK-2800 Kgs. Lyngby, Denmark; § 1569 Bayer AG, Crop Science Division, Environmental Safety, D-40789 Monheim, Germany; ∥ substitute ApS, Graaspurvevej 55, 2400 Copenhagen, Denmark; ⊥ Department for Evolutionary Ecology and Environmental Toxicology, Goethe University, 60438 Frankfurt am Main, Germany; # Department of Environmental Sciences, College of Agriculture and Environmental Sciences, University of South Africa, Florida 1710, Roodepoort, South Africa

**Keywords:** insecticides, seed application, spray application, xylem and phloem flux, plant uptake modeling, pollinator exposure

## Abstract

Seed coatings protect agricultural crops from pests, but they can expose pollinators to residues. We extended a dynamic model for pesticide uptake from soil into maize plants with a flower compartment, including nectar and pollen. Field experiments with seed/soil and spray applications were simulated successfully. Calibrated loss rates usually exceeded dissipation rates empirically fitted to declining concentrations due to continuous delivery of chemicals seen by the model (soil to plant components). Simulated dissipation consists of growth dilution and degradation (nonvolatile compounds) with half-lives fitted for imidacloprid in pollen of 0.2 to 0.9 days, close to observations following spray application. Our model predicts that mobile, persistent, and nonvolatile chemicals are potentially translocated to pollen if present in soil. This is relevant also for persistent, mobile, and toxic (PMT) chemicals released from reclaimed wastewater or via sewage sludge application. Our model can be incorporated into existing frameworks to estimate the exposure of pollinating insects.

## Graphical abstract

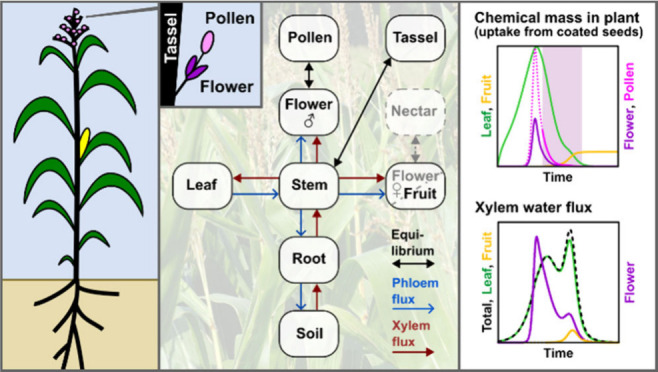

## 1 Introduction

All annual field crops are angiosperms, which means they develop flowers with pollen and bear seeds in fruits.^1^ Flowers can attract insects, whether for nectar or/and pollen foraging or for other reasons: a broad variety of insect species visit flowers.^2^ Plant protection against pests is essential in conventional agriculture. Insecticides soluble in water can be sprayed on or incorporated into soil, from where they can be taken up with the transpiration water by plant roots and translocated into all plant components to protect them against insect attacks. A more targeted application method for insecticides or fungicides is seed coating. Seed treatment with xylem-mobile (systemic) insecticides allows protection of crops from harmful insects with small amounts of active ingredients and thus avoids repeated spraying of fields and spray drift to nontarget areas. Important insecticides applied as seed coating are neonicotinoids, such as imidacloprid, clothianidin, thiamethoxam, acetamiprid, thiacloprid, and dinotefuran.^3^ Most of these insecticides are highly toxic to pests by targeting nicotinic acetylcholine receptors (similar to nicotine, which explains the name), under certain circumstances persistent over the vegetation period in soil and plant, and sufficiently water-soluble and hydrophilic to be translocated within the xylem to leaves, flowers, and fruits.^4^ However, it was recognized early that systemic pesticides may also be translocated into nectar,^5^ and hence, the often persistent and widely applied neonicotinoids were suspected to lead to poisoning of pollinators, such as honey bees.^6^

On a global scale, seed treatment is currently widely applied, though a few active ingredients are banned in some regions now, for example, in the European Union, where the application of the neonicotinoids imidacloprid, clothianidin, and thiamethoxam was strictly limited to indoor treatments and emergency use.^7^ Moreover, in 2023 the European Food Safety Agency EFSA released a guidance document which requires exposure and toxicity assessment of pesticides for bees.^8^ In this guidance, exposure of bees via nectar and pollen to insecticides originating from seed coating is calculated from empirical RUD values. A generic one-compartment prediction model for uptake of pesticides into nectar and pollen was derived by Li.^9^ The model consists of an analytical solution of a first-order linear differential equation and constant average conditions as input. The author recommended that “multi-compartment uptake models, weather conditions in addition to temperature and humidity, and agricultural activities be considered in future studies”. There is thus a knowledge gap, as no precise deterministic prediction tool for the time-dynamic concentration pattern of residues in pollen and nectar after seed or soil treatment is currently available.

Beyond assessing pesticides in the context of pollinator risk assessment and environmental safety, impacts associated with pesticide use are also relevant to evaluate the environmental behavior or chemical footprint of products and technologies, including agricultural practices, using life-cycle assessment (LCA) as an ISO-standardized methodology.^10,11^ While human toxicity and ecotoxicity impacts from pesticides and other chemicals are commonly included in existing life cycle impact assessment (LCIA) methods^12,13^ and assessed in various LCA studies (e.g., refs 14–16 ), pesticide-related impacts on pollinating insects are currently missing in most available methods.^17^ While ecotoxicity effect test data for pollinating insects are becoming increasingly available, related exposure is rarely measured and relies on estimates that allow linking pesticide applications to residual masses in nectar and pollen. However, a major challenge for introducing pollinator impacts in LCA is related to estimating exposure to pesticide residues in pollen and nectar as an important exposure pathway,^18^ mainly because available experimental data do not cover the wider range of pesticides and exposure settings, while existing plant uptake models do usually not include pollen and nectar as receiving exposure compartment (e.g., refs 19–22 ). Developing a dedicated plant uptake model that allows for simulating the dynamics of pesticide uptake into pollen and nectar as a starting point for estimating pollinator exposure is hence a current research gap to advance on by considering pollinator impacts also in the context of LCA. This can be done, for example, by directly combining the results of such a plant uptake model with pesticide uptake by pollinators and relevant effect test data to link pollinator impacts back to pesticide use.

The model used in this study is based on four compartments coupled by growth-dependent and time-variable fluxes in the xylem and phloem. This concept was established in 2011 by Rein et al.^19^ and applied, tested, and validated in a range of studies.^21–24^ Here, we introduce a new flower compartment to the model, in order to simulate the translocation of chemicals from soil through xylem and phloem to flowers, nectar, and pollen. The model was then applied to predict pesticide residues in the pollen of maize (*Zea mays* L.), and the model output was compared with experimental results. Field experiments with four insecticides were modeled, including seed/soil applications (imidacloprid, thiacloprid, and tetraniliprole) and foliar spray application (spiromesifen).

## 2 Materials and Methods

### 2.1 Experimental Data

Data from field and greenhouse experiments with maize and various pesticides were obtained from Bayer AG, including time series of concentrations measured in different plant matrices (leaf, tassel, flower, pollen), guttation fluid, and soil. Details of the simulated field experiments are provided in the Supporting Information (SI), Table S1.

### 2.2 Plant Input Data

Data on growing maize stem, leaf, and fruit mass were obtained from field experiments carried out near Aydin, Turkey.^25^ Root mass as a function of time was estimated from growing stem mass, assuming that root mass is 60% of stem mass (based on observations by Prof. Dr. Cafer Turgut, Adnan Menderes University Aydin, Turkey, at a site close to the experimental field used in the study of Koca and Erekul;^25^ personal communication). These observations were obtained under Mediterranean climate conditions, with a growth period (sowing to harvest) of 128 days. Since the modeled pesticide experiments relate to longer growth periods of about 150 days, the growth observations of Koca and Erekul^25^ were linearly scaled (Table S2). Simulation of growing plant mass was done by fitting logistic growth curves (cf. Section S2). Fitted growth rate constants *k*_gr_, lag times before the first appearance *t*_lag_ (relevant for fruit, flower, tassel, pollen), as well as assumed or fitted initial masses *M*_0_ and final masses *M*_max_ are shown in Table S3. Observed versus simulated plant mass as a function of time is displayed for root, stem, leaf, and fruit in Figure S1 and for flower, tassel, and pollen in Figure S2.

The model setup in Rein et al.^24^ allowed considering the growth of individual fruits. This concept was extended further in the present study to consider individual appearance and growth for all plant parts as an option, with

1 $$\frac{dM_{p,i}}{dt}=\{\begin{array}{l} k_{\mathrm{gr},p,i}\cdot M_{p,i}\cdot \left(1-\frac{M_{p,i}}{M_{\max,p,i}}\right)\;\mathrm{if}\;t\geq t_{\mathrm{lag},p,i}\\ 0\;\mathrm{if}\;t<t_{\mathrm{lag},p,i} \end{array}$$

2 $$\frac{dM_{p}}{dt}=\overset{n_{p}}{\underset{i=1}{\sum }}\frac{dM_{p,i}}{dt}$$

where *M* is plant mass, index p stands for the plant compartment (fruit, leaf, etc.), and index i stands for the individual plant compartment part (individual fruits, leaves, etc.). Parameter *k*_gr,p__,i_ is the first-order growth rate constant and *M*_max,p,__.i_ is the final plant mass (at harvest). Equation 1 indicates the change of individual plant part mass with time; eq 2 describes the change of total plant part mass (all fruits, all leaves, etc.) with time, where *n*_p_ is the number of individual plant parts (number of fruits, leaves, etc.). Herein, *t*_lag,p__,i_ indicates the lag phase before and between the appearance of individual plant parts.

For the calculation of xylem flux, transpiration was coupled to growth.^19^ Growing plant mass induces transpiration *Q* ^Xy^ (L/d), which is the water flux within the xylem of roots and stem:

3 $$Q^{\mathrm{Xy}}=T_{C}\cdot \overset{N}{\underset{j=1}{\sum }}\frac{dM_{j}}{dt}$$

where *T*_*C*_ (L/kg) is the transpiration coefficient (50 L/kg-fresh weight (fw) considered for maize^26^) and *M*_j_ (kg-fw) is the mass of above-ground plant parts j (stem, leaf, fruit, flower). Xylem and phloem fluxes were considered between soil, root, stem, leaf, flower and fruit (Figure 1). Xylem water fluxes to leaf, fruit, and flower (*Q*_L_^Xy^, *Q*_F_^Xy^, *Q*_Fl_^Xy^) were derived by averaging with the respective surface areas (section S3). Phloem flux from leaf to stem and further downward to root and soil *Q*_LstR_^Ph^ (L/d) was assumed to be 5% of xylem flux.^27^ Phloem flux from leaf to stem to fruit *Q*_LStF_^Ph^ (L/d) and from leaf to stem to flower *Q*_LStFl_^Ph^ (L/d) was obtained from growing fruit and flower mass (cf. section S3).

**Figure 1:**
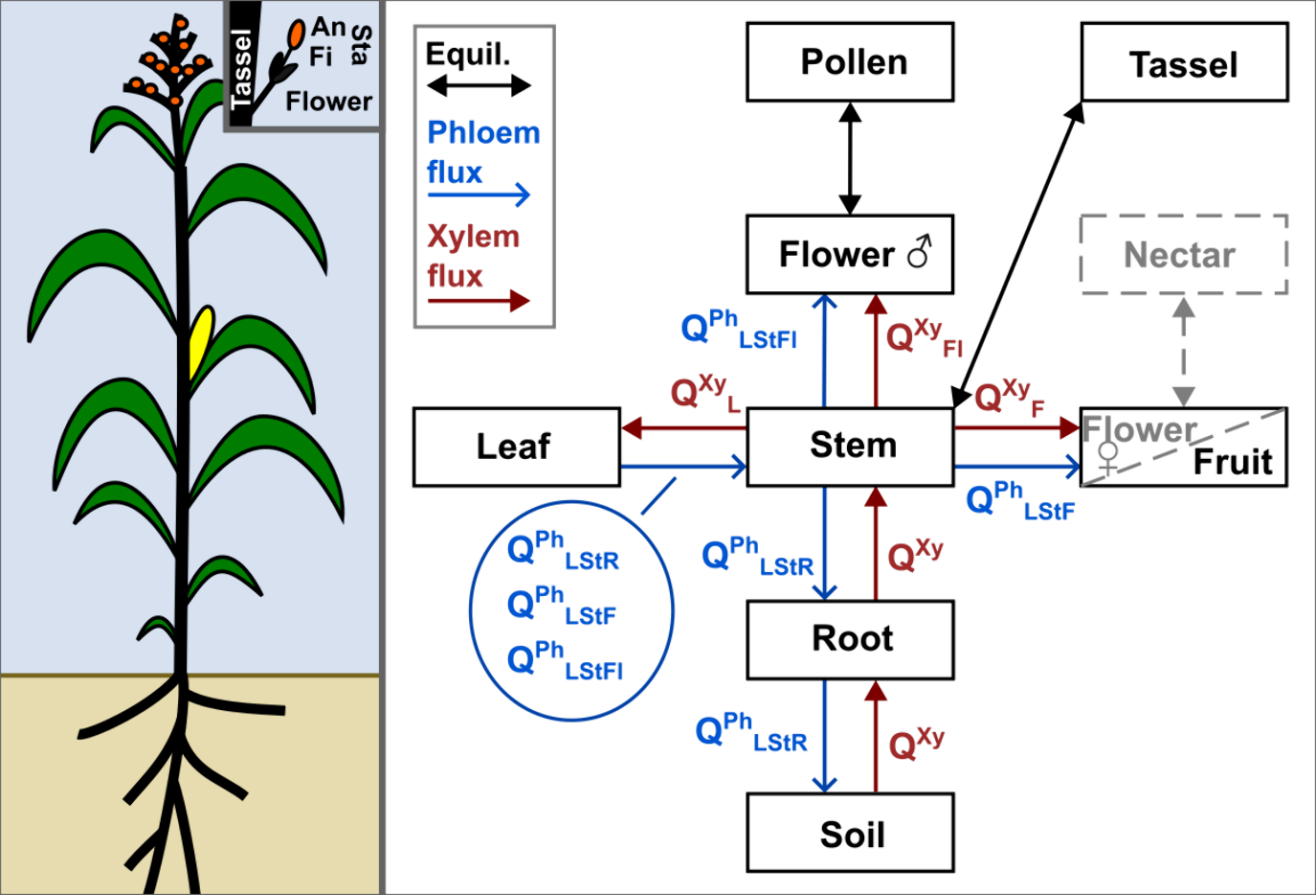
Maize plant (stamen Sta with filament Fi and anther An which contains pollen sacs) and compartments considered in the dynamic model approach. *Q* ^Xy^: xylem flux in root and stem; *Q*_L_^Xy^, *Q*_F_^Xy^, and *Q*_Fl_^Xy^: xylem flux in leaf, fruit and flower, respectively; *Q*_LStR_^Ph^: phloem flux from leaf to stem and root; *Q*_LStF_^Ph^: phloem flux from leaf to stem and fruit; *Q*_LStFl_^Ph^: phloem flux from leaf to stem and flower.

### 2.3 Modeling of Chemical Plant Uptake

The dynamic model approach of Rein et al.^24^ with the compartments soil, roots, stem, leaves, and fruits was extended with the compartments flower, tassel, pollen, and nectar, as illustrated in Figure 1. Equilibrium partitioning was assumed between the tassel and stem and between the flower and pollen. Equations were also set up for nectar in equilibrium with (female) flowers but were not further considered in this study, since maize does not produce nectar. The chemical mass balance equations (set up for soil, roots, stem, leaves, fruits, and flowers) are summarized in Section S3 of the Supporting Information (SI). Equilibrium partitioning assumptions are described below.

#### 2.3.1 Flowers and Tassel

The new equation for the change of chemical mass in flower with time is

4 $$\frac{dm_{\mathrm{Fl}}}{dt}=-k_{\mathrm{Fl}}m_{\mathrm{Fl}}-\left(\frac{f_{n}^{\mathrm{Fl}}A_{\mathrm{Fl}}P_{\mathrm{Fl}}10^{3}}{K_{\mathrm{FlW}}}\right)C_{\mathrm{Fl}}+\left(\frac{Q_{\mathrm{Fl}}^{\mathrm{Xy}}}{K_{\mathrm{St}\mathrm{Xy}}}+\frac{Q_{\mathrm{LStFl}}^{\mathrm{Ph}}}{K_{\mathrm{StPh}}}\right)C_{\mathrm{St}}+I_{\mathrm{Fl}}$$

where *m*_Fl_ (mg) and *C*_Fl_ (mg/kg) are chemical mass and concentration in flower (concentration *C* is obtained as chemical mass *m* divided by compartment mass *M*). *C*_St_ (mg/kg) is chemical concentration in stem, *Q* ^Xy^_Fl_ (L/d) is xylem flux to flowers, and *Q* ^Ph^_LStFl_ (L/d) is phloem flux from leaf to stem and further to flowers. *k*_Fl_ (d^–1^) is a first-order rate constant for biodegradation and other unaccounted losses (such as wash-off). The second term on the right-hand side describes loss to the atmosphere, and *I*_Fl_ is external input to flowers, including atmospheric deposition emissions such as spray application of pesticides. *K*_StXy_ and *K*_StPh_ are equilibrium partition coefficients (L/L) stem to xylem and stem to phloem, respectively (cf. section S3), and *K*_FlW_ is the equilibrium partition coefficient between flower and water (described below).

Maize plants develop male flowers on tassels that grow on the stem tip. Concentration in tassel (T) was assumed to be in equilibrium with concentration in stem (St), with partition coefficients tassel to water (*K*_TW_) and stem to water (*K*_StW_):

5 $$C_{T}=C_{\mathrm{St}}\cdot \frac{K_{\mathrm{TW}}}{K_{\mathrm{StW}}}$$

In the model approach, the flower compartment was connected directly to the stem to avoid numerical problems (small tassel volume, high flow-through).This does not affect calculated mass balances and is justified because the concentration in tassel approaches equilibrium anyway very quickly.

#### 2.3.2 Nectar and Pollen

Insecticides applied via seed treatment are usually xylem mobile. These insecticides, for example imidacloprid, are thus mainly transported with the transpiration stream to the flower. The xylem ends in the apoplast, and the water distributes in the apoplastic intracellular space, from where it evaporates into the atmosphere (most of the water translocated in the transpiration stream is never taken up into the cells). All parts of the flower are in contact with the same apoplast, and we therefore expect the same chemical activity (truly dissolved or free concentration in water) everywhere in the flower. This is the underlying assumption for the calculation of concentrations in nectar and pollen from the concentration in flower. For ionizing chemicals, the concentration ratio between total flower Fl (including water W, lipids L, and proteins prot) to the water within the flower is described separately for the neutral (n) and ionic (i) fraction of the chemical:

6a $$K_{\mathrm{FlW},n}=W_{\mathrm{Fl}}+L_{\mathrm{Fl}}\cdot a\cdot K_{\mathrm{OW},n}^{b}+{\mathrm{prot}}_{\mathrm{Fl}}\cdot K_{\mathrm{HSA}}$$

6b $$K_{\mathrm{FlW},i}=W_{\mathrm{Fl}}+L_{\mathrm{Fl}}\cdot a\cdot K_{\mathrm{OW},i}^{b}+{\mathrm{prot}}_{\mathrm{Fl}}\cdot K_{\mathrm{HSA}}$$

In the above equations, indexes n and i refer to the neutral and ionized species, respectively. *K*_OW,n_ (L/L) corresponds to common *K*_OW_, and *K*_OW,i_ is estimated as log *K*_OW,i_ = log *K*_OW,n_ – 3.5.^28^ Coefficient *K*_HSA_ (L/kg, with L of water per kg of protein) describes partitioning between human serum albumin and water, used as a proxy for protein to water partitioning.^29^ Factor *a* equals 1.22 L/kg (1/ρ_octanol_) and *b* (−) is a correction exponent for differences between plant lipids and octanol, and is 0.95.^30^ The partition coefficients for neutral and ionic fractions are then added, weighted with their respective fractions:

6c $$K_{\mathrm{FlW}}=f_{n}\cdot K_{\mathrm{FlW},n}+f_{i}\cdot K_{\mathrm{FlW},i}$$

where *f* is the fraction of neutral *n* and ionic *i* chemical in water at given pH as described below (pH apoplast is used as reference). For neutral compounds, *K*_FlW_ is obtained from eq 6a.

The freely dissolved concentration follows then from the total concentration as

7 $$C_{W,\mathrm{Fl}}=\frac{C_{\mathrm{Fl}}}{K_{\mathrm{FlW}}}$$

Nectar is produced from phloem sap alone or from phloem and xylem sap in the nectary cells in the flowers and is excreted into the extracellular space.^31^ Here, it comes in contact and mixes with the apoplast water originating from the xylem, and water evaporating from the nectar can be replenished from the apoplast. Xylem sap is sour (pH 5.5, Table S4), and nectar typically also has such a low pH.^32^

Therefore, we keep the assumption that nectar is in equilibrium with the apoplast of the flower. Nectar can be seen as water containing sugar, but carbohydrates have a modest adsorption capacity, similar to that of water,^33^ which we neglect therefore. Then

8 $$C_{\mathrm{Ne}}=C_{W,\mathrm{Fl}}=\frac{C_{\mathrm{Fl}}}{K_{\mathrm{FlW}}}$$

with chemical concentration in nectar *C*_Ne_, in water of the flower *C*_W,Fl_, and in flower *C*_Fl_. Pollen is also assumed to be in equilibrium with the water in the flower apoplast, so that concentration in pollen *C*_Po_ can be related to *C*_W,Fl_ or to *C*_Fl_ as

9 $$C_{\mathrm{Po}}=C_{W,\mathrm{Fl}}\cdot K_{\mathrm{PoW}}=C_{\mathrm{Fl}}\cdot \frac{K_{\mathrm{PoW}}}{K_{\mathrm{FlW}}}$$

For ionizing chemicals, adsorption of both neutral and ionic molecules was calculated with eqs 10b and 10c and added according to their fractions at given pH:

10a $$K_{\mathrm{PoW}}=f_{n}\cdot K_{\mathrm{PoW},n}+f_{i}\cdot K_{\mathrm{PoW},i}$$

with

10b $$K_{\mathrm{PoW},n}=W_{\mathrm{Po}}+L_{\mathrm{Po}}\cdot a\cdot K_{\mathrm{OW},n}^{b}+{\mathrm{prot}}_{\mathrm{Po}}\cdot K_{\mathrm{HSA}}$$

10c $$K_{\mathrm{PoW},i}=W_{\mathrm{Po}}+L_{\mathrm{Po}}\cdot a\cdot K_{\mathrm{OW},i}^{b}+{\mathrm{prot}}_{\mathrm{Po}}\cdot K_{\mathrm{HSA}}$$

For neutral compounds, *K*_PoW_ is obtained from eq 10b.

Fractions of the neutral (*f*_n_) and ionized (*f*_i_) species are calculated as^28,29^

11 $$f_{n}=1/\left(1/\gamma_{n}+D_{S}/\gamma_{d}\right)$$

12 $$f_{i}=f_{n}\cdot D_{S}$$

*D*_S_ is the activity ratio between the neutral and ionic species, obtained from the Henderson–Hasselbalch equation as

13 $$D_{S}=10^{\alpha \cdot \left(pH_{s}-pK_{a}\right)}$$

where α is 1 for acids and −1 for bases, pH_S_ is soil pH, and p*K*_a_ is the acid or base dissociation constant. γ_n_ and γ_d_ are the activity of the neutral and dissociated species in soil, respectively:

14a $$\log\gamma_{n}=k_{\mathrm{Se}}\cdot I$$

14b $$\log\gamma_{d}=-Az^{2}\left(\sqrt{I}/\left(1+\sqrt{I}\right)-0.2\cdot I\right)$$

where *k*_Se_ is the Setchenov coefficient (0.3), *I* is ionic strength (assumed to be low in soil, with *I* = 0.01 M), *A* is 0.5 (for conditions at 20 °C and 1 atm pressure), and *z* is the valency or charge number.

In summary, for ionizable chemicals, the Cell Model (describing chemical dynamics within plant cells^28,29,34,35^ ) was applied to obtain partition coefficients in root, stem, leaf, fruit, and flower (cf. section S3). Once the chemical reached the flower, the cell model was not used again. Instead, equilibrium distribution to the apoplastic intracellular space was assumed (eqs 6a and 10a), as described above. An alternative solution would be using the cell model also for the partitioning of chemicals into pollen, based on the assumption that the chemical is distributed in the flower through the symplast (within the cells), where it has to cross at least one cell membrane to reach pollen. Then, dissociation and membrane transport may additionally affect concentrations in pollen. Three ionizable compounds were considered in this study. At the given pH range in soil and plant cells (cf. Tables S4 and S5), the dissociated fractions were negligible for imidacloprid, but not for tetraniliprole and thiacloprid (p*K*_a_ values of 11.1, 9.0, and 7.0, respectively, Table S6).

#### 2.3.3 Solution of ODEs

The chemical mass balances in the compartments soil, roots, stem, leaves, flowers, and fruits are described by an ordinary differential equation (ODE) for each compartment. The six ODEs are shown in section S3. The model approach was set up within Matlab R2024b, where solver ode15s for stiff matrices was used for integrating the whole differential equation system. Pesticide application was mimicked by pulse input to different compartments, depending on the scenario. Input to soil is assumed for seed coating (where only a very small fraction is assumed to be taken up into the seed) and for soil spray/incorporation, or input to different plant parts for foliar spray application. Least-squares fitting of predicted to measured concentrations was performed with manual expert adjustment of model parameters in an iterative procedure. Fitting parameters included the (unknown) individual input to soil and the above-ground plant compartments (stem, leaf, fruit, flower) as well as the first-order rate constants for loss by biodegradation and wash-off *k* (d^–1^) in soil and plant compartments (section S3).

In addition to the loss rates used in numerical modeling, first-order dissipation rate constants *k*_diss_ (d^–1^) were fitted to available measurements and to simulated concentrations for describing overall concentration decrease (Tables S7 and S8). This was done to compare empirically observable net dissipation (which is due to a combination of processes such as biodegradation, growth dilution, and wash-off, but also gain by continuous translocation) with single processes, and with other observations from the literature. Half-lives were calculated from dissipation rate constants as *t*_1/2_ = ln(2)/*k*_diss_.

## 3 Results and Discussion

Figure 2 shows simulated imidacloprid mass (a–c) and concentration (d–f) as a function of time in soil, root, stem (a, d); leaf, fruit, flower (b, e); and tassel and pollen (c, f). A field experiment with seed treatment was modeled, with an application of 13.5 mg per m^2^ of the active ingredient to the seeds at the time of sowing. Input to soil and loss rate constants for all compartments were among the unknowns and had to be estimated by curve fitting. Different combinations of input to soil and loss from soil can lead to a successful fit of the measured concentrations in leaves, tassel, and pollen. Due to the absence of measured concentrations in soil, no decision about the correct combination for soil could be made. The fitted input to soil was 1.5 mg per m^2^, if loss from soil was set to zero. If input to soil was set equal to the applied amount (13.5 mg/m^2^), the loss rate constant in soil was fitted to 0.04 d^–1^. The latter approach was considered further, as a more likely assumption. Other loss rate constants were fitted to 0.1 d^–1^ for stem, 0.25 d^–1^ for leaf, 2 d^–1^ for flower, and 0 for root and fruit (Table S7, M1).

**Figure 2:**
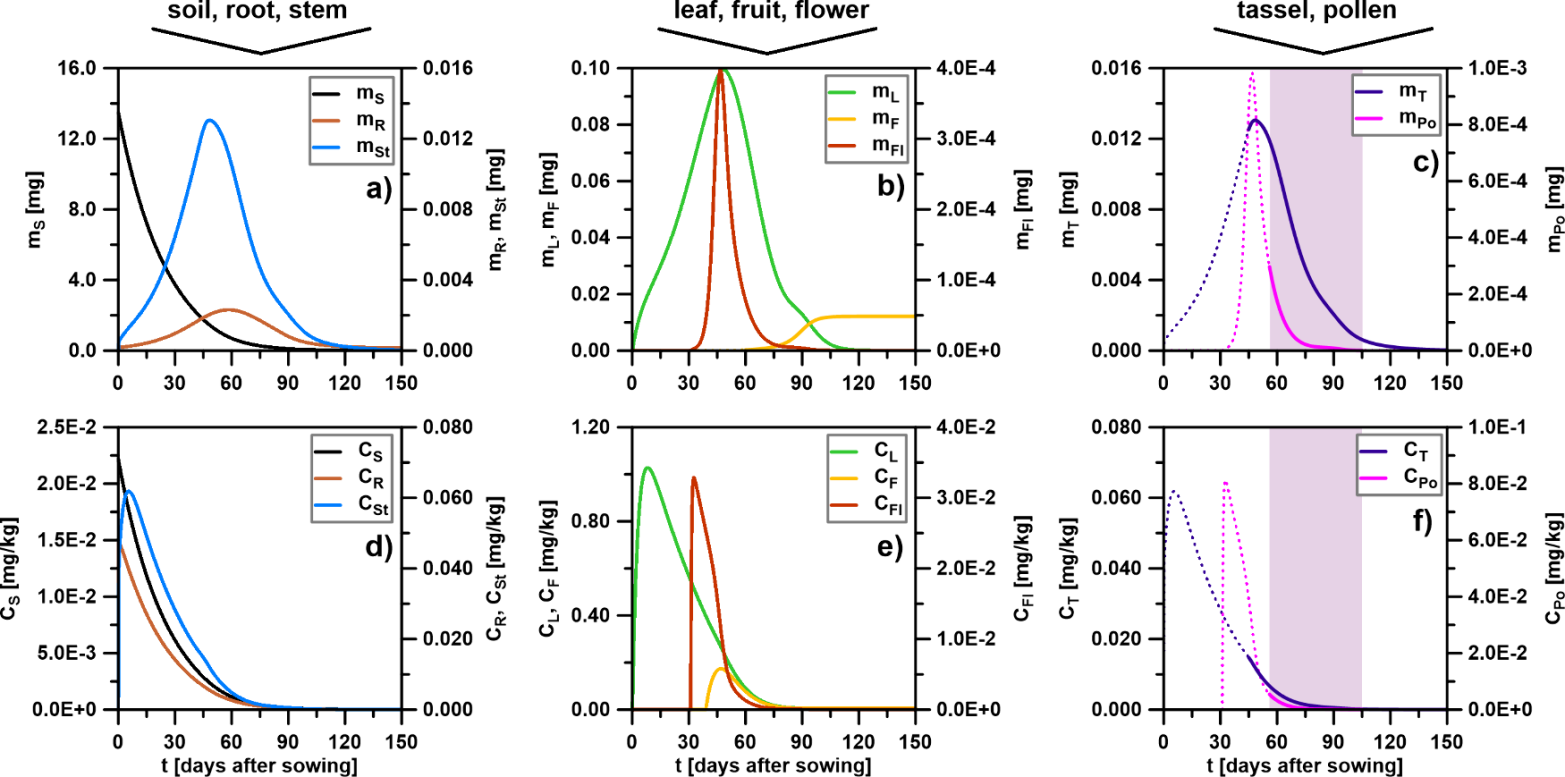
Simulated mass (a, b, c) and concentration (d, e, f) of imidacloprid as a function of time in soil and maize plant compartments. Pink areas (c, f) indicate estimated pollen shed period. Dotted lines: chemical mass (c) concentration (f) before tassel and pollen appearance.

Figure 2ab shows the chemical mass in all dynamically coupled compartments (soil, root, stem, leaf, fruit, and flower), as described in eqs S1–S6. The flow of xylem (Figure 3b) is calculated from plant growth (Figure 3a), and the transport of imidacloprid from soil to root, stem, and leaves is with the xylem flow. Hence, the steepest increase of chemical mass within roots and stems is seen during the intense (exponential) growth phases, followed by a decline later on, as depicted in Figure 2a. The same steep increase of chemical mass during intense growth followed by a decline is seen for leaves, flowers, and fruits (Figure 2b). Concentrations in tassel and pollen were calculated using equilibrium to stem (eq 5) and to flower (eq 7). Stem and tassel have the same composition (Table S3), thus *K*_TW_ equals *K*_StW_, and concentrations in tassel equal those in stem (Figure 2df).

**Figure 3:**
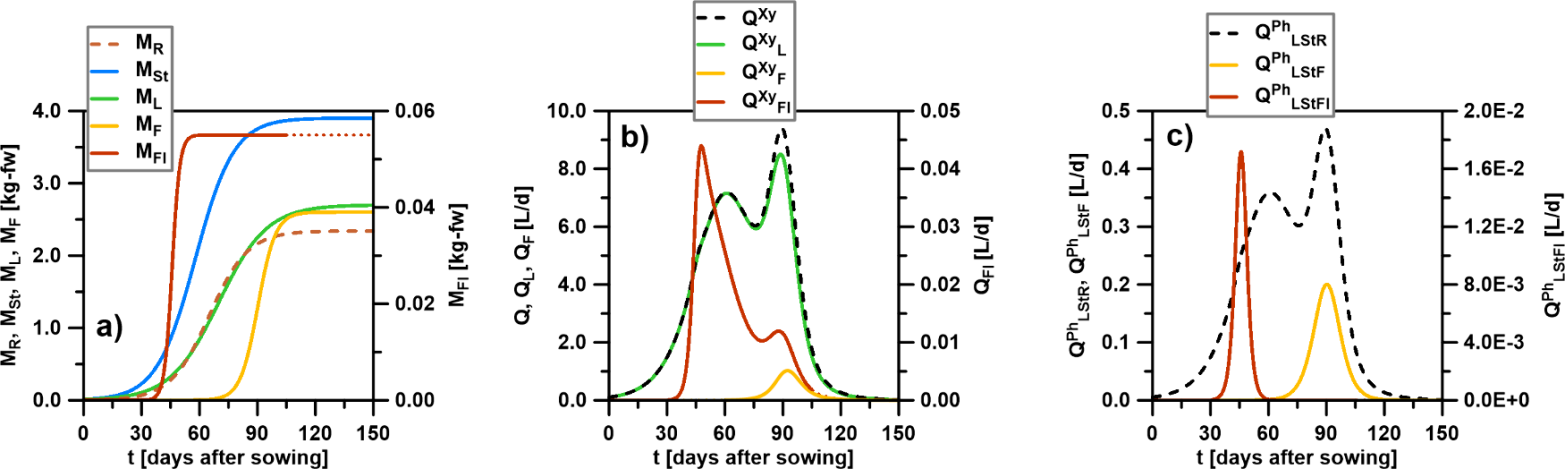
Simulated plant compartment masses (a), xylem fluxes (b), and phloem fluxes (c) as a function of time. Dotted line (a): before flower appearance.

The highest concentrations appear generally early, long before the peak mass is reached, for stem at *t* = 10 d, leaves at *t* = 15 d, flowers shortly after *t* = 30 d, and fruits at *t* = 50 d (Figure 2de). This happens because the ratio of flux to plant mass is the highest at the beginning of the exponential growth phase, when plant masses are still rather small. Tassel and pollen are in chemical equilibrium to stem and flower, and therefore, chemical mass within them closely follows those in stem and flower, respectively (Figure 2c). Concentrations in flower, pollen, and fruit are thus highest *before* their full appearance, following the model calculations. However, flowers do not appear out of nothing: they form from the apical meristem, which is already present in the seeds. Once the signal is given, this apical meristem divides until buds are formed, from which the flowers appear. Tassels and flowers appear after 45 to 50 days after sowing (DAS), pollen between 56 and 105 DAS, and fruits approximately 60 DAS and last until harvest (Figures S1 and S2). The highest concentrations were calculated for the time early in this process, again because the ratio of flux to volume is largest at the beginning of the growth phase. At this early time, the flowers are not yet open, and the pollen cannot be sampled nor the fruits. Therefore, chemical masses and concentrations calculated for these plant organs before their appearance are displayed as dotted lines in Figures 2 and 3: the calculated concentrations are considered real, but the respective plant parts cannot be sampled, and the calculated concentrations therefore cannot be verified.

The time frame for insects visiting flowers to collect pollen depends on the local meteorological conditions, the maize variant, and the availability of other crops and plants in the area considered. In the presented field studies, this time frame was between 56 and 105 DAS (assumed end of pollen shedding), as indicated by the pink areas in Figure 2. Only within this period can exposure of pollinating insects to contaminated pollen at maize plants occur. Samples from leaves, tassels, and pollen were taken in this time period and analyzed for imidacloprid. The observed concentrations match the simulated ones overall quite well (Figure 4).

**Figure 4:**
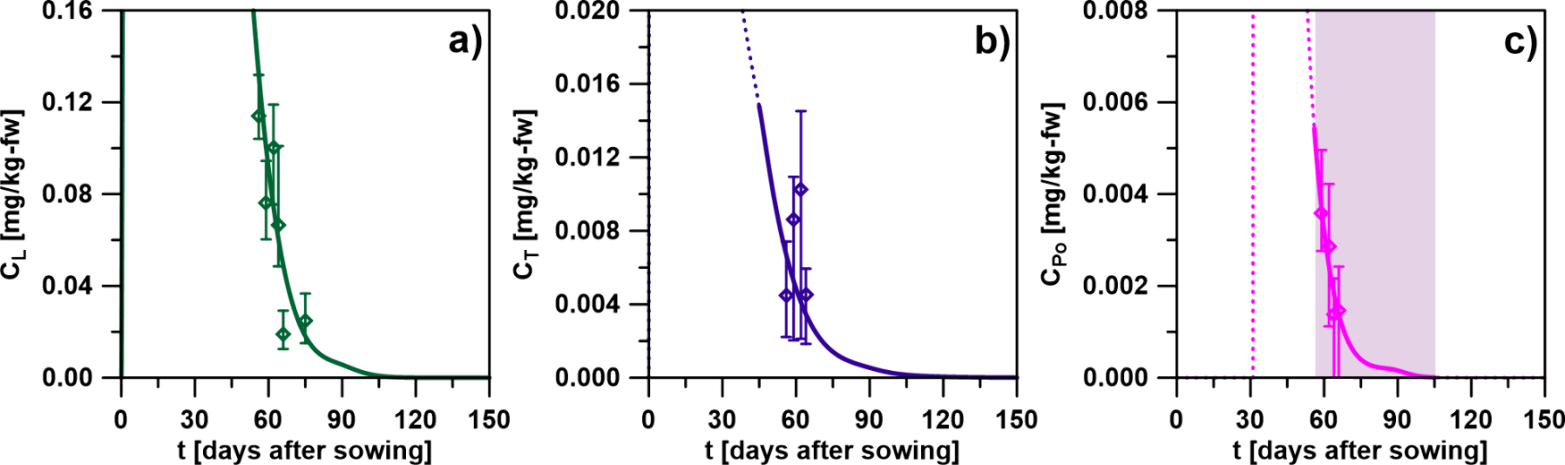
Observed (symbols) versus simulated (curves) imidacloprid concentration as a function of time in leaf (a), tassel (b), and pollen (c). Average observed concentrations, error bars indicate minimum and maximum (3–5 replicates).^36^ Pink area in panel c indicates estimated pollen shed period. Simulated concentrations are zoomed in for better visibility, i.e., comparison to observations (cf. Figure 2e,f for the full curves).

Details on fitted parameters and statistical curve fit evaluation can be found in Table S7 (experiment M1). Further experiments with imidacloprid and seed treatment could be simulated well, too, as shown in Figure S3. Chemical input to soil is assumed to correspond to the mass applied via seed coating (as discussed above) for the nine experiments (M1–M9): fitted loss constants *k* are 0.03–0.04 d^–1^ for soil; 0.05–0.1 d^–1^ for stem; 0.25–0.8 d^–1^ for leaf; 0.8–2.0 d^–1^ for flowers; and 0 for soil, root, and fruit (Table S7).

Imidacloprid observations in pollen that result from application in the previous year could also be explained by modeling (Figure S4 and Table S7, experiments SG and Y). However, uncertainties are associated with the estimated initial mass in soil (residues from previous application; no measurements of soil concentrations reported) and in the case of experiment Y also with observed pollen concentrations below the level of quantification (LOQ).

Pollen concentrations of thiacloprid, resulting from seed treatment, can be described by the model as well, as shown in Figure S5. Measurements were however below LOQ, leading to considerable uncertainty. The applied mass of the active ingredient was 10 mg per m^2^ for both experiments, and assuming this value for the input to soil led to simulated pollen concentrations that reach LOQ, at the time of observation. Fitted loss rate constants are 0.1 d^–1^ for stem and leaf; 0.8 d^–1^ for flowers; and 0 for soil, root, and fruit (Table S8).

From field experiments with foliar spray of spiromesifen, observed concentrations in tassel and pollen are reported.^37^ Those could be described well by modeling, matching observed concentration dynamics (Figure S6). Estimated input was to stem; to leaves; and, by low portions, to flowers (Table S8). Observations were not available for leaves, and input to leaves was insensitive to tassel and pollen concentrations. Loss rate constants were found to be 0.03–0.1 d^–1^ for stem; 0.1 d^–1^ for leaf; 0.2–0.3 d^–1^ for flowers; and 0 for soil, root, and fruit (Table S8).

Finally, field experiments with tetraniliprole were modeled, as presented in Figure S7. Those involved chemical application via soil incorporation and soil spray. However, observed pollen concentrations were below the level of detection (LOD) for experiment N and below the LOQ for experiment SB, so that simulation results and fitted parameters have to be interpreted with caution. A much higher input to soil was assumed in the model (50 and 55 mg per m^2^) compared to the reported incorporated and sprayed mass (15 and 15.3 mg per m^2^), to approach the reported LOD in pollen (Figure S7, upper row). For the experiment with soil spray, soil and pollen concentrations were measured. With an estimated input to soil of 55 mg per m^2^, measured soil concentrations were met, and simulated pollen concentrations were close to the reported LOQ. Loss rate constants were fitted 0.1 d^–1^ for stem and leaf; 0.2 d^–1^ for flower; and 0 for soil, root, and fruit (Table S8).

### 3.1 Residue per Unit Dose, RUD

The application rate of imidacloprid (seed coating) in the simulated experiments is 11.9 to 13.5 mg per m^2^ (Table S7). Twelve mg per m^2^ corresponds to 0.12 kg/ha, and maximum residue levels in pollen of about 0.012 mg/kg fw were calculated within the pollen shed period (Figure S3), and 0.001 to 0.012 mg/kg fw were measured. This corresponds to a residue per unit dose (RUD) of 0.0083 to 0.1 mg/kg per kg applied on one hectare. These RUD are very low, compared to the usual RUD of insecticides via spray application which for pollen is in median 26 mg/kg:kg/ha.^38^ This underlines that seed coating is a method of insecticide application that can minimize the exposure of nontarget organisms. These RUD values range from 0.002 to 0.2875 mg/kg for pollen (90th percentile 0.061 mg/kg) and from 0.0024 to 0.13 mg/kg for nectar. Bonmatin et al.^39^ found imidacloprid in the pollen of maize crops. However, a serious drawback is that insecticide residues were found in all plant parts over the whole growth period (Figure 2) so that the time window for potential exposure of visiting or consuming nontarget organisms is longer than with spray application. Seed coating brings the active ingredient into direct contact with the roots, which leads to efficient uptake. It is, in this respect, comparable to drip irrigation. Zhang and Li^40^ compared the efficiency of foliar spray or broadcast application with drip irrigation and found that drip irrigation produced a higher application efficiency. The drawback is, like with seed coating, that this high efficiency leads to high levels of residues in the harvested edible plant components, which can result in increased food safety concerns.

### 3.2 Dissipation versus Loss and Chemical Delivery

Empirical dissipation rate constants (*k*_diss_), calibrated to the overall concentration decrease in the plant compartments, are lower than the loss rate constants found using the numerical model (*k*). This can be explained by continuous delivery of chemicals from soil via roots and stems further to leaves and flowers, which acts in the model simulation as negative dissipation (gain) but cannot be observed by analyzing a plant sample.

Empirical dissipation half-lives (calculated from *k*_diss_) in flower and pollen range from 5.3 to 6.9 days for imidacloprid (Table S7), those for thiacloprid and tetraniliprole from 5.3 to 5.8 and 5.8 to 6.3 days, respectively. Spiromesifen has the shortest dissipation half-life, with 2.3 to 3.5 days (Table S8). These empirical dissipation times are up to ten times larger than those listed by EFSA for dissipation after spray application. Based on 39 observations from 12 pesticides in five crops, a range of half-lives in pollen from 0.18 to 4.3 days and with median at 0.63 days was reported.^41^ The empirical dissipation half-lives listed in Tables S7 and S8 do not consider that chemical mass lost from flower and pollen is replaced by new chemical mass translocated via xylem. However, simulated dissipation rates do capture this effect. Simulated dissipation is composed of growth dilution (with growth rate up to 0.5 d^–1^), degradation and wash-off (imidacloprid fitted loss rates 0.8 to 3.5 d^–1^, Table S7), and volatilization (negligible for imidacloprid). The sum of these processes leads to a half-live of 0.17 to 0.53 days (0.2 to 0.9 days without growth), and thus similar to those obtained for spray application, where no continuous delivery from soil happens. Thus, for very mobile insecticides (imidacloprid, tetraniliprole, thiacloprid; Table S6) and application via seed treatment, empirical dissipation half-lives fitted to measured (or simulated) concentrations lead to underestimation of the real loss, because the gain due to translocation cannot be considered. Spiromesifen, which is applied by spray application and is translocated only at very low rates due to the high adsorption (Table S6), has the lowest empirical dissipation half-times in Tables S7 and S8, only 2.3 to 3.5 d. Similar observations were made by Jacobsen et al.,^42^ who used a pesticide fate model for the quantification of dissipation processes. Uptake of compounds from soil into plants was shown to lead to a negative dissipation process, i.e., a gain. For methomyl, the most polar chemical of their data set with log K_OW_ of 0.09, uptake from soil reduced the loss rate from fruits by −33%; while for cyfluthrin, the chemical with the highest log K_OW_ of 6, no change of dissipation rate due to translocation from soil to fruits was found.

Our modeling studies reveal that an adequate description of chemical fluxes, driven by xylem and phloem flows, is essential for understanding the chemical uptake and translocation within the plant. This includes chemical transport from soil (seed coating) via roots and stem to leaves and flowers, where the delivery of chemicals to different plant compartments is balanced with loss due to degradation; growth dilution; and, for volatile compounds, volatilization. In combination with equilibrium assumptions (stem to tassel and flower to pollen), this consideration allows a plausible description of chemical concentration in plant tissue (including leaves, tassel, pollen) as a function of time.

### 3.3 Comparison with Other Studies

The modeling result can be compared to experimental studies on the uptake of imidacloprid from coated seeds and translocation to pollen. Donnarumma et al.^43^ measured the distribution of imidacloprid in soil and its translocation into maize plant. Seeds were dressed with 1 mg imidacloprid per seed (12 seeds and thus about 12 mg per m^2^), close to the applied amount reported in this study (Table S1). Concentrations in pollen and kernels at harvest (130 d) were <1 μg/kg, which is below the range observed and simulated here (Figures 4, S3, and S4). The authors concluded “that maize pollen source should not be relevant for acute toxicity impact on honey bees”.

The same result, concentration in pollen below 1 μg/kg or below detection limit, was obtained by Choudhary and Sharma^44^ for mustard pollen. Schmuck et al.^45^ found imidacloprid in sunflower pollen at a concentration of 3.9 μg/kg after seed dressing with 0.7 mg peer seed. Schöning and Schmuck^46^ measured in rape, sunflower and maize pollen residue concentrations of imidacloprid of 5 μg/kg after seed dressing with 0.7 mg peer seed. Laurent and Rathatao^47^ detected imidacloprid in pollen of sunflower, following seed treatment, with similar concentration to floret dish and other parts of the flower but much lower than in leaves. Bonmatin et al.^48^ found residue concentrations of imidacloprid after seed treatment in sunflower pollen between 1 and 11 μg/kg, with median 3 μg/kg, and no imidacloprid was detected in 17% of the samples (LOD 0.3 μg/kg). In maize pollen, the average level was 2.1 μg/kg, however, with high variation. More studies are reviewed and listed in Zioga et al.^49^ Given the cited examples, the concentrations on imidacloprid reported in this study are within the level reported in other experimental studies.

Skerl et al.^50^ reported concentrations of thiacloprid in apple pollen after spray application of 90 μg/kg the day after application, corresponding to a RUD of 0.94 mg/kg per kg/ha. In contrast, seed treatment (10 mg per m^2^) did not lead to measurable residues of thiacloprid in pollen (Figure S5). Measured residue concentrations of spiromesifen in pollen after spray application on mustard were as high as 2.1 mg/kg at the day of application, but declined to 0.72 mg/kg within 1 day and were below detection limit (50 μg/kg) after 10 days.^44^ Corresponding RUD values were 9.34 mg/kg per kg/ha at day 0, 3.20 mg/kg per kg/ha at day 1, and <0.0002 mg/kg per kg/ha after 10 days. This is very similar to the pattern in our simulation (Figure S6), where maximum concentrations of spiromesifen were about 1.5, 5, and 10 mg/kg (RUD 5 to 25 mg/kg per kg/ha) but declined rapidly within a few days.

The EFSA 2013 guidance^51^ lists in Appendix F empirical RUD values for residues in nectar and pollen following seed treatment in the unit mg/kg nectar or pollen per mg applied on single seed. These RUD values range from 0.002 to 0.2875 mg/kg for pollen (90th percentile 0.061 mg/kg) and from 0.0024 to 0.13 mg/kg for nectar. Bonmatin et al.^39^ found imidacloprid in the pollen of maize treated with seed dressing of 1 mg/seed ranging from 0.3 μg/kg (LOD) to a maximum of 18 μg/kg with an average level of 2.1 μg/kg, corresponding to an RUD pollen of 0.002 mg/kg. The concentrations in pollen simulated with our model and with 1 mg of imidacloprid per seed (12 to 13 seeds per m^2^) are at peak 0.035 mg/kg-fw but decline to <0.005 mg/kg-fw during the pollen shed period and are thus within the range found by EFSA,^51^ but to the lower end (Figure 4c), and similar to the findings of Bonmatin et al.^39^

Our model further predicts translocation of imidacloprid into nectar. This is not relevant for maize plants that do not produce nectar but has been confirmed for many other crops, and the repeated detection of neonicotinoids in nectar as well as in pollen was reported.^52^

A simpler model approach for uptake of pesticides into nectar and pollen was applied by Li.^9^ A one-compartment model was employed to calculate uptake from soil into leaf, and partitioning between leaves, nectar, and pollen was calculated according to Chiou et al.,^33^ with log K_OW_, lipid content, and fraction of carbohydrates as input parameters. This model does, however, not consider the complex interaction of dynamic plant growth and xylem/phloem flow on chemical uptake and translocation, which is crucial for understanding chemical concentration as a function of time in nectar and pollen. These complex processes are considered in our model approach, developed in this study.

In summary, in this study, we show for the first time a comparison of measured and modeled insecticide residues in pollen. The agreement between both in level and time demonstrates that it is possible to simulate insecticide uptake into roots following seed treatment and subsequent translocation to flowers and pollen in phloem and with the transpiration stream. Of the four compounds studied, only imidacloprid and spiromesifen were detected in levels above LOQ. Imidacloprid is also the most polar of the four substances at pH 7 and essentially nonvolatile (low log *D* and *K*_AW_, cf. Table S6). In xylem, at pH 5, imidacloprid and thiacloprid have log *D* values of <1, while tetraniliprole is slightly less polar (log *D* in xylem at pH 5 to 4 is 1.6 to 1.5). Spiromesifen (log K_OW_ = 5.25) is not applied via seed treatment in agricultural practice but solely by foliar spray application. In simulations with 735 different pesticides in peanut plants, all lipophilic substances with log K_OW_ > 5 showed negligible translocation within plants, but most were well-suited for foliar spray application.^53^ On the other hand, all mobile, persistent, and nonvolatile chemicals are potentially translocated to pollen if present in soil or as coating on seeds, as predicted by the plant model developed in this study. This is not limited to soil- or seed-applied pesticides: a number of organic chemicals have been detected in surface waters.^54^ Up to 30% of those fulfill the suggested criteria for labeling as vPvM (very persistent, very mobile) or as PMT (persistent, mobile, and toxic).^55–57^ River and surface waters such as reclaimed wastewaters are increasingly used for irrigation of field crops. It is likely that any mobile and persistent chemicals are translocated from the soil to upper plant parts, including pollen. For example, pharmaceuticals originating from reclaimed wastewater have been widely detected in irrigated field crops.^58^ There is a high probability that these chemicals also find their way to pollen and end up in bees and other pollen-consuming insects. Yet, there is little known about the potential effects. Recently, Nightingale et al.^59^ detected a number of emerging contaminants (pharmaceuticals and other compounds) in honey, most of which were polar chemicals.

The presented model for the simulation of pesticide uptake into nectar and pollen can be incorporated into existing frameworks to estimate the exposure of pollinating insects. This can be an important component of risk assessment, life cycle impact assessment, and chemical footprinting of pesticides and PMT chemicals. Pollinator exposure can be combined with respective toxicity effects, aiming for safe and sustainable pest management and agricultural practices. Our model can support pesticide design and experimental exposure assessments, where it can efficiently generate time-dependent data on residue levels in the plant, including nectar and pollen.

## Supplementary Material



## Notes

Section S1 provides details of the modeled field experiments; S2 describes maize growth observations and simulations; S3 gives details of the applied modeling approach; S4 provides physicochemical properties, soil and plant input data; S5 presents simulation results for individual experiments (PDF)

## Abbreviations

DAS: days after sowing
EFSA: European Food Safety Authority
LCA: life cycle assessment
LCIA: life cycle impact assessment
LOD: level of detection
LOQ: level of quantification
ODE: ordinary differential equation
PMT: persistent, mobile, and toxic chemicals
RUD: residue per unit dose
vPvM: very persistent, very mobile chemicals
