# Supplementary material for: Mechanistic Model for Simulating Pesticide Uptake into Maize Pollen
Source: J Agric Food Chem. 2026 May 28;74(22):17025–34. doi: 10.1021/acs.jafc.6c02800 (PMC13266987; doi:10.1021/acs.jafc.6c02800)
Supplement: Supplementary file 1 [file jf6c02800_si_001.pdf]

Supporting Information

Mechanistic model for simulating pesticide uptake into maize pollen

Arno Rein[1,*], Stefan Trapp[2], Klaus Hammel[3], Peter Fantke[4,5,6]

[1] Chair of Hydrogeology, TUM School of Engineering and Design, Technical University of Munich, Arcisstr. 21, D-80333 Munich, Germany

[2] Department of Environmental and Resource Engineering, Technical University of Denmark, Bygningstorvet 115, DK-2800 Kgs. Lyngby, Denmark

[3] Bayer AG, Crop Science Division, Environmental Safety, D-40789 Monheim, Germany

[4] substitute ApS, Graaspurvevej 55, 2400 Copenhagen, Denmark

[5] Department for Evolutionary Ecology and Environmental Toxicology, Goethe University, 60438 Frankfurt am Main, Germany

[6] Department of Environmental Sciences, College of Agriculture and Environmental Sciences, University of South Africa, Florida 1710, Roodepoort, South Africa

* Corresponding author, arno.rein@tum.de

Table of contents

This Supporting Information has 18 pages, in total, including:

S1 Field experimental data (including Table S1)	Page S-2
S2 Maize growth observations and simulations (including Figures S1 and S2 and Table S2)	Page S-2
S3 Details of the applied modeling approach	Page S-4
S4 Plant and soil input data, physicochemical properties (including Tables S3 to S6)	Page S-7
S5 Simulation results for individual experiments (including Figures S3 to S7 and Tables S7 and S8)	Page S-9
List of Supporting Information references	Page S-15

S1 Field experimental data

Table S1: Details of the modeled field experiments.

Active ingredient	Application type	Applica-tion rate (mg/m^2)	Concentration measured in:	Location	Reference
Imidacloprid a	Seed treatment	13.5	L, T, Po	USA (~20 km SSW' Kansas City)	Miller et al.[1]
Imidacloprid a	Seed treatment	13.5	L, T, Po	USA (~20 km SSW' Kansas City)	Miller et al.[1]
Imidacloprid a	Seed treatment	12.8	L, T, Po	USA (~20 km SSW' Kansas City)	Miller et al.[1]
Imidacloprid a	Seed treatment	13.2	L, T, Po	USA (~200 km NW' Kansas City)	Miller et al.[1]
Imidacloprid a	Seed treatment	13.2	L, T, Po	USA (~200 km NW' Kansas City)	Miller et al.[1]
Imidacloprid a	Seed treatment	13.5	L, T, Po	USA (~200 km NW' Kansas City)	Miller et al.[1]
Imidacloprid a	Seed treatment	12.7	L, T, Po	USA (~225 km NW' Kansas City)	Miller et al.[1]
Imidacloprid a	Seed treatment	12.7	L, T, Po	USA (~225 km NW' Kansas City)	Miller et al.[1]
Imidacloprid a	Seed treatment	11.9	L, T, Po	USA (~225 km NW' Kansas City)	Miller et al.[1]
Imidacloprid	None e	n.a.	S, Po, Gf	France (~10 km W' Orléans)	Ythier et al.[2]
Imidacloprid	None e	n.a.	S, Po, Gf	France (~100 km NNW' Lyon)	Schöning & Gerhardt[3]
Thiacloprid b	Seed treatment	10	Po	Germany (~10 km E' Würzburg	Schöning[4]
Thiacloprid b	Seed treatment	10	Po	Germany (~10 km E' Würzburg	Schöning[4]
Tetraniliprole c	Soil incorporation	15	Po, Gf	France (~50 km N' Lyon)	Noel et al.[5]
Tetraniliprole c	Soil spray	15.3	S, Po, Gf	Germany (~20 km W' Cologne)	Striffler & Ballhaus[6]
Spiromesifen d	Foliar appl. f	27.7	T, Po	USA (~170 km E' Toronto)	Gould and Jerkins[7]
Spiromesifen d	Foliar appl. f	29.3	T, Po	USA (~400 km WSW' Kansas City)	Gould and Jerkins[7]
Spiromesifen d	Foliar appl. f	28	T, Po	USA (~25 km WSW' Kansas City)	Gould and Jerkins[7]

a: imidacloprid FS 600 (active ingredient, a.i., 600 g/L), b: thiacloprid FS 400 (a.i. 400 g/L), c: tetraniliprole SC 200 (a.i. 200 g/L), d: spiromesifen SC 240 (a.i. 240 g/L), e: treatment in previous year, f: foliar application (one spray at growth stage BBCH61); n.a.: not applicable; L: leaf, T: tassel, Po: pollen, S: soil, Gf: guttation fluid.:

S2 Maize growth observations and simulations

Growth observations by Koca and Erekul[8] were linearly scaled from 128 days to 150 day, as shown in Table S2. Figure S1 shows observed versus simulated root, stem, leaf and fruit mass as a function of time. Investigated growth of flower, tassel and pollen (Figure S2) is based on data and observations of Liu et al.[9] and Sun et al.[10] (parameters summarized below). Simulation of growing plant mass was done by fitting logistic growth curves (cf. section S1). Fitted growth rate constants k_{gr}, assumed or fitted initial masses M_0 and final masses M_{max} as well as lag times before the first appearance t_{lag} (flower, tassel, pollen) are included in Table S5 for root, stem, leaf, fruit and flower. Parameters used for simulating tassel and pollen growth (Figure S2 b, e, c, f) are as follows: M_0 for tassel and pollen as for fruits (Table S5); M_{max} of 0.26 and 0.03 kg-fw per m^2, k_{gr} of 0.4 and 1.0 d^{-1}, respectively. Since equilibrium partitioning assumptions were used (stem to tassel and flower to pollen), tassel and pollen growth was not implemented in the model (cf. Methods section).

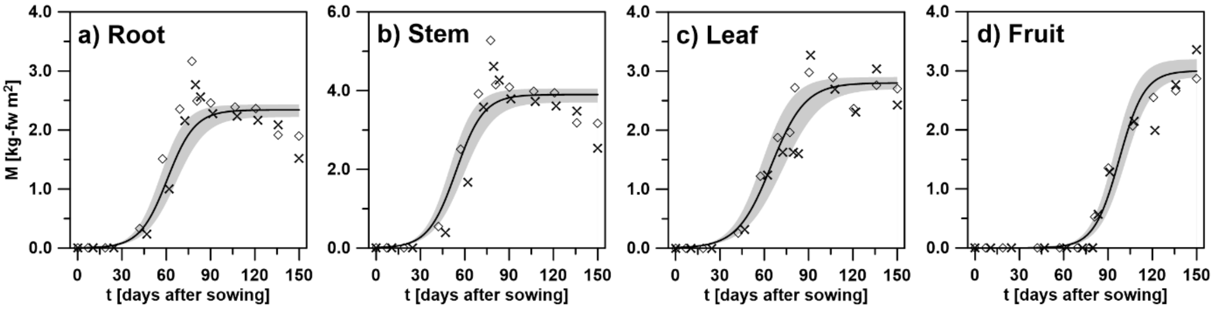

Figure S1: observed (symbols, two field experiments) versus simulated (curves) mass of root (a), stem (b), leaf (c) and fruit (d). Scaled from 130 to 150 days (cf. Table S2). black curve: best estimate, gray areas: upper/lower estimate range.

Table S2: Time of growth stages (i) in the field experiments of Koca and Erekul[8], relating to a growth period of 128 days, versus (ii) scaled to a growth period of 150 days. So: sowing; Em: emergence; t_{obs}: time of observation; t_{scaled}: scaled time; DAS: days after sowing.

Growth stage	So	Em	V4	V8	V12	V16	VT	R1	R2	R3	R4	R5	R6
t_{obs} (DAS)	0	6	16	36	49	59	66	69	77	91	103	116	128
t_{scaled} (DAS)	0	7.0	18.8	42.2	57.4	69.1	77.3	80.9	90.2	106.6	120.7	135.9	150

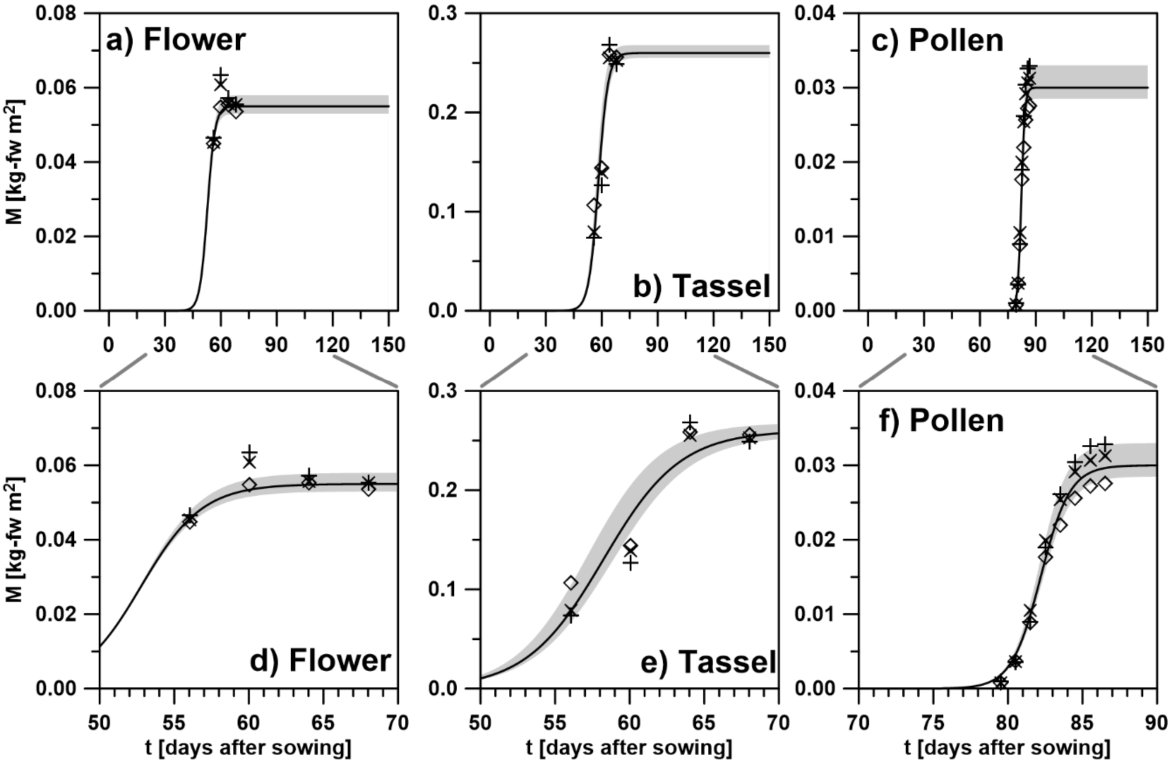

Figure S2: observed (three field trials; symbols) versus simulated mass (curves) of flower (a, d), tassel (b, e) and pollen (c, f).

S3 Details of the applied modeling approach

The model approach of Rein et al.[11] was extended by the compartments flower, tassel and pollen. The chemical mass balance equations were set up as described below (Equation S1-S6). They include the six compartments soil, root, stem, leaf, fruit and flower. In addition, chemical concentration was calculated in the two compartments tassel and pollen. This was done based upon equilibrium assumptions, as described in the main manuscript (Equation 5 and 7).

$$\frac{dm_S}{dt} = -k_S m_S - \left(Q_r K_{WS} + Q_l K_{WS} + Q^{Xy} K_{XyW} K_{WS} f_c + f_n^S A_S P_S \rho_{Sw} 10^3\right) C_S + \frac{Q_{LStR}^{Ph}}{K_{RPh}} C_R + I_S \quad \text{(S1)}$$

$$\frac{dm_R}{dt} = -k_R m_R - \left(\frac{Q^{Xy}}{K_{RXy}} + \frac{Q_{LStR}^{Ph}}{K_{RPh}} + \frac{f_n^R A_R P_R 10^3}{K_{RW}}\right) C_R + Q^{Xy} K_{XyW} K_{WS} f_c C_S + \frac{Q_{LStR}^{Ph}}{K_{StPh}} C_{St} + I_R \quad \text{(S2)}$$

$$\frac{dm_{St}}{dt} = -k_{St} m_{St} - \left(\frac{Q_L^{Xy} + Q_F^{Xy} + Q_{Fl}^{Xy}}{K_{StXy}} + \frac{Q_{LStR}^{Ph} + Q_{LStF}^{Ph} + Q_{LStFl}^{Ph}}{K_{StPh}} + \frac{f_n^{St} A_{St} P_{St} 10^3}{K_{StW}}\right) C_{St} + \frac{Q^{Xy}}{K_{RXy}} C_R + \frac{Q_{LStR}^{Ph} + Q_{LStF}^{Ph} + Q_{LStFl}^{Ph}}{K_{LPh}} C_L + I_{St} \quad \text{(S3)}$$

$$\frac{dm_L}{dt} = -k_L m_L - \left(\frac{Q_{LStR}^{Ph} + Q_{LStF}^{Ph} + Q_{LStFl}^{Ph}}{K_{LPh}} + \frac{f_n^L A_L P_L 10^3}{K_{LW}}\right) C_L + \frac{Q_L^{Xy}}{K_{StXy}} C_{St} + I_L \quad \text{(S4)}$$

$$\frac{dm_F}{dt} = -k_F m_F - \left(\frac{f_n^F A_F P_F 10^3}{K_{FW}}\right) C_F + \left(\frac{Q_F^{Xy}}{K_{StXy}} + \frac{Q_{LStF}^{Ph}}{K_{StPh}}\right) C_{St} + I_F \quad \text{(S5)}$$

$$\frac{dm_{Fl}}{dt} = -k_{Fl} m_{Fl} - \left(\frac{f_n^{Fl} A_{Fl} P_{Fl} 10^3}{K_{FlW}}\right) C_{Fl} + \left(\frac{Q_{Fl}^{Xy}}{K_{StXy}} + \frac{Q_{LStFl}^{Ph}}{K_{StPh}}\right) C_{St} + I_{Fl} \quad \text{(S6)}$$

In these equations, m_i (mg) is chemical mass in compartment i (soil S, root R, stem St, leaf L, fruit F, flower Fl) and t (d) is time. Chemical concentration C_i (mg/kg) is obtained as $C_i = m_i / M_i$, with compartment mass M_i (kg). k_i are first-order rate constants for loss by biodegradation and wash-off. Q_l and Q_r are flows of leaching and surface water runoff, respectively (L/d). f_c (-) is a correction factor that is important for very polar compounds (log $K_{OW} < -2$) to account for a reduced root uptake (for other compounds $f_c = 1$). The uptake of such compounds into roots is slower than the uptake of water, so that f_c can be calculated as $f_c = P_{R,comp} / P_{R,water}$ (with $f_c < 1$). This reflects a different root membrane permeability for a compound and for water ($P_{R,comp}$ (m/s) and $P_{R,water}$ (m/s), respectively). In literature, f_c is also referred to as plant uptake factor *PUF,* retardation factor *R* or correction factor *F*.[12-14]

The mass flow rate of constant external chemical input I_i (mg/d) consists of atmospheric input (C_A (mg/m^3) is chemical concentration in air) plus external constant emission $I_{em,i}$ to each compartment (such as anthropogenic emission). It is given for soil (index S) as

$$I_S = \left[\frac{f_n^S A_S P_S \rho_{Sw} 10^3}{K_{AW} K_{WS}} \cdot (1 - f_p) + A_S v_{dep} \cdot f_p\right] \cdot C_A + I_{em,S} \qquad \text{(S7)}$$

and for plant compartment j (root R, stem St, leaf L, fruit F, flower Fl) as

$$I_j = \left[\frac{f_n^j A_j P_j}{K_{AW}} \cdot (1 - f_p) + A_j v_{dep} \cdot f_p\right] \cdot C_A + I_{em,j} \qquad \text{(S8)}$$

It is assumed that only the neutral fraction (f_n) of ionizable compounds can volatilize, considered as f_n^S, f_n^R, f_n^{St}, f_n^L, f_n^F and f_n^{Fl} in Equation (S1-S7) and as f_n^j in Equation (S8) (details see below). For the modeling of neutral compounds, these factors are set to 1. Logistic growth was assumed for the plants, i.e. for the mass M_j (kg) of above-ground plant compartments j (stem, leaf, fruit, flower). Transpiration Q^{Xy} (L/d) is induced as long as the plant is growing, as described in the main manuscript (Eq. 1-3). Q^{Xy} relates to the water flux within the xylem of the roots and the stem. The water flux into leaves, fruits and flowers Q_L, Q_F and Q_{Fl} can be derived by averaging with the respective surface areas A_L, A_F and A_{Fl} (m^2). For neutral compounds, where concentrations in xylem and phloem sap are equal, phloem flux from leaves via stem to fruits Q^{Ph}_{LStF} and phloem flux from leaves via stem to flowers Q^{Ph}_{LStFl} can be subtracted for Q^{Xy}_L, while Q^{Ph}_{LStF} can be added for Q^{Xy}_F and Q^{Ph}_{LStFl} can be added to Q^{Xy}_{Fl} (applying the approach of Trapp[15] and Rein et al.[16] and extending it for flower):

$$Q_L^{Xy} = \mathrm{Q} \cdot \frac{A_L}{A_L + A_F + A_{Fl}} - Q_{LStF}^{Ph} - Q_{LStFl}^{Ph} \qquad \text{(S9)}$$

$$Q_F^{Xy} = \mathrm{Q} \cdot \frac{A_F}{A_L + A_F + A_{Fl}} + Q_{LStF}^{Ph} \qquad \text{(S10)}$$

$$Q_{Fl}^{Xy} = \mathrm{Q} \cdot \frac{A_F}{A_L + A_F + A_{Fl}} + Q_{LStFl}^{Ph} \qquad \text{(S11)}$$

with Q^{Ph}_{LStF} = dM_F/ dt x (1-W_F) x $T_{C,Ph}$ and Q^{Ph}_{LStFl} = dM_{Fl}/ dt x (1-W_{Fl}) x $T_{C,Ph}$, where dM_F/ dt (kg/d) and dM_{Fl}/ dt (kg/d) are the change of the fruit and flower mass, respectively, W is water content (in fruit F and flower Fl) and $T_{C,Ph}$ (L/kg) is phloem flux per fruit mass, where a value of 10 L per kg-dw (dry weight) was used.[15] Plant compartment surface areas A_j (m^2), corresponding to those exposed to the atmosphere, are obtained from multiplying plant mass M_j (kg) with the specific surface area (m^2/kg) for each plant compartment. Phloem flux from leaves via stem to root (and finally to soil) was calculated as Q^{Ph}_{LStR} = p_{PhXy} x Q, where p_{PhXy} (-) is the ratio of phloem to xylem flux (assumed 0.05 [15, 16]). Permeability P (m/s), needed to describe diffusive flux between soil or plant and the atmosphere (used in Equation S1-S8), was obtained as described by Trapp and Matthies[17] and Rein et al.[16] K_{AW} (L/L) is the air to

water partition coefficient and K_{WS} (kg/L) the water to soil partition coefficient obtained as $K_{WS} = \rho_{S,wet} / (f_{OC} \times K_{OC} \times \rho_{S,dry} + \theta_W + \theta_A \times K_{AW})$ with wet and dry soil density $\rho_{S,wet}$ and $\rho_{S,dry}$ (kg/L), respectively, soil organic carbon content f_{OC} (kg-OC/kg), organic carbon to water partition coefficient K_{OC} (L/kg-OC), soil water and air content θ_W (L/L) and θ_A (L/L), respectively. Wet soil density is given as $\rho_{S,wet} = \rho_{S,dry} + \theta_W \rho_W$ with water density ρ_W (1 kg/L).

For neutral lipophilic compounds, K_{OC} can be estimated using the octanol to water partition coefficient K_{OW} (L/L): log K_{OC} = 0.81 x log K_{OW} + 0.1.[18] Franco and Trapp[19] developed regressions to estimate the K_{OC} of acids and bases, where the following equations can be used for weak acids: log $K_{OC,n}$ = 0.54 x log K_{OW} + 1.11 for the neutral species and log $K_{OC,d}$ = 0.11 x log K_{OW} + 1.54 for the anion. The following equations can be used for weak bases: log $K_{OC,n}$ = 0.42 x log K_{OW} + 1.34 for the neutral species and log $K_{OC,d}$ = 0.47 x log K_{OW} + 1.95 for the cation. Total $K_{OC} = f_{n,S} \times K_{OC,n} + f_{i,S} \times K_{OC,i}$, where $f_{n,S}$ and $f_{i,S}$ are the fractions of the neutral and ionized species in soil. These are calculated as a function of soil pH, the acid dissociation constant pK_a and the ionic strength of soil solution as described by Trapp[20].

Chemical dynamics of ionizable compounds within plant cells (cytoplasm, vacuole, xylem, phloem) were simulated with the Cell Model, which we have set up according to Trapp[20, 21], Trapp and Horobin[22] and Trapp et al.[14]. This Cell Model was coupled to the mass balance equations (Equation S1-S6). It calculates the fractions of the neutral and dissociated chemical species in the different plant parts (f_n and f_d) and partition coefficients (L/L) between cytoplasm and water K_{CytW}, vacuole and water K_{VacW}, xylem and water K_{XyW}, as well as phloem and water K_{PhlW}. Those were obtained by solving the differential equations for the change of chemical mass in the organelles (chemical fluxes) analytically and referring resulting concentrations (in cytoplasm, vacuole, xylem and phloem) at steady-state to concentration in water (details see Trapp and Horobin[22]).

For ionizing substances, to account for the different pH-values in cytosol and vacuole, the plant to water partition coefficients K_{RW}, K_{StW}, K_{LW} , K_{FW}, K_{FlW} (except for the apoplast pathway that was considered for pollen, see below and main manuscript) and K_{TW} (root, stem, leaf, fruit, flower and tassel to water) needed in Equation (S1-S6) were obtained as $K_{PlantW} = (K_{CytW} V_{Cyt} + K_{VacW} V_{Vac}) / (V_{Cyt} + V_{Vac})$, where V is volume of the vacuole (index Vac) and the cytoplasm (index Cyt). Information on the parameterization of the Cell Model is provided in Table S6. Partition coefficients (L/kg) K_{jXy} (plant compartment to xylem) and plant to phloem K_{jPh} were calculated as $K_{RXy} = K_{RW}/K_{XyW}$, $K_{StXy} = K_{StW}/K_{XyW}$, $K_{TXy} = K_{TW}/K_{XyW}$, $K_{RPh} = K_{RW}/K_{PhlW}$, $K_{StPh} = K_{StW}/K_{PhlW}$, $K_{LPh} = K_{LW}/K_{PhlW}$, $K_{TPh} = K_{TW}/K_{PhlW}$. For pollen, the apoplast pathway was considered (cf. equations in the main manuscript). For neutral compounds, plant to water partition coefficients were obtained as $K_{jW} = W_j + L_j \times 1.22 \times K_{OW}^{b,j} + prot_j \times K_{HSA}$ with water content W, lipid content L, coefficient b and protein $prot$ for plant compartment j. K_{HSA} is used as a proxy for protein to water partitioning (cf. main manuscript for details).

S4 Plant and soil input data, physicochemical properties

Table S3: Maize input data. For plant mass, 8 plants per m^2 are considered.

Parameter	Symbol	Unit	Value	Source
**Root**				
Root water content	W_R	L/kg	0.89	a
Root lipid content	L_R	g/g	0.025	b
Root protein content	$prot_R$	g/g	0.02	c
Correction factor, root lipids vs. oct.	b_R	-	0.77	d
Root growth rate	$k_{gr,R}$	d^{-1}	0.10	e
Initial root mass per m^2	$M_{0,R}$	kg fw	0.0035	f
Final root mass per m^2	$M_{max,R}$	kg fw	2.34	g
**Stem**				
Stem water content	W_{St}	L/kg	0.79	h
Stem lipid content	L_{St}	g/g	0.02	d
Stem protein content	$Prot_{St}$	g/g	0.02	i
Correction factor, stem lipids vs. oct.	b_{St}	-	0.95	d
Specific stem area	$SStA$	m^2 kg^{-1} fw	0.379	j
Stem growth rate	$k_{gr,St}$	d^{-1}	0.10	k
Initial stem mass per m^2	$M_{0,St}$	kg fw	0.012	f
Final stem mass per m^2	$M_{max,St}$	kg fw	3.90	k
**Leaf**				
Leaf water content	W_L	L/kg	0.75	h
Leaf lipid content	L_L	g/g	0.02	d
Leaf protein content	$Prot_L$	g/g	0.05	i
Correction factor, leaf lipids vs. oct.	b_L	-	0.95	d
Specific leaf area	SLA	m^2 kg^{-1} fw	6.92	l
Leaf growth rate	$k_{gr,L}$	d^{-1}	0.09	k
Initial leaf mass per m^2	$M_{0,L}$	kg fw	0.010	f
Final leaf mass per m^2	$M_{max,L}$	kg fw	2.70	k
**Fruit**				
Fruit water content	W_F	L/kg	0.86	h
Fruit lipid content	L_F	g/g	0.04	m
Fruit protein content	$Prot_F$	g/g	0.09	n
Correction factor, fruit lipids vs. oct.	b_F	-	0.95	d
Specific fruit area	SFA	m^2 kg^{-1} fw	1.07	o
Lag time of fruit appearance	$t_{lag,F}$	d	39	k
Fruit growth rate	$k_{gr,F}$	d^{-1}	0.22	k
Initial fruit mass per m^2	$M_{0,F}$	kg fw	3.3 x 10^{-5}	f
Final fruit mass per m^2	$M_{max,F}$	kg fw	2.60	k
**Flower**				
Flower water content	W_{Fl}	L/kg	0.75	p
Flower lipid content	L_{Fl}	g/g	0.02	p
Flower protein content	$Prot_{Fl}$	g/g	0.02	q
Correction factor, flower lipids vs. oct.	b_{Fl}	-	0.95	p
Specific flower area	$SFlA$	m^2 kg^{-1} fw	0.379	e
Lag time of flower appearance	$t_{lag,Fl}$	d	31	r
Flower growth rate	$k_{gr,Fl}$	d^{-1}	0.5	r
Initial flower mass per m^2	$M_{0,Fl}$	kg fw	3.3 x 10^{-5}	s
Final flower mass per m^2	$M_{max,Fl}$	kg fw	0.055	r
**Tassel**				
Tassel water content	W_T	L/kg	0.79	e
Tassel lipid content	L_T	g/g	0.02	e
Tassel protein content	$Prot_T$	g/g	0.02	q
Correction factor, tassel lipids vs. oct.	b_T	-	0.95	e
**Pollen**				
Pollen water content	W_{Po}	L/kg	0.52	u
Pollen lipid content	L_{Po}	g/g	0.035	v
Pollen protein content	$prot_{Po}$	g/g	0.14	v
Correction factor, pollen lipids vs. oct.	b_{Po}	-	0.95	e

a: value for wheat assumed [16, 23]; b: Trapp[24]; c: average from Song et al.[25]; d: Trapp and Matthies[17]; e: value of stem assumed; f: based on values for dry weight for wheat (Rein et al.[16]); g: estimated as 60% of stem weight (average percentage based on sampled root and stem mass, field experiments with corn maize, Prof. Dr. Cafer Turgut, personal communication); h: average value within growing stages V16, VT and R1 (Koca and Erekul [8]), corresponding to the time of measurements in tassel, flower and pollen; i: FeedTables [26], Shu et al.[27]; j: based on Yu-kui et al.[28]; k: fitted from observations of Koca and Erekul[8]; l: based on Zhou et al.[29], Danalatos et al.[30]; m: average from Barrera-Arellano et al.[31]; n: based on FAO[32]; o: based on Iqbal et al.[33], Sangamithra et al.[34]; p: values of leaf considered; q: value of stem considered; r: fitted to observations of increasing floret numbers (Liu et al.[9]) and estimated floret mass (Sun et al.[10], Fonseca et al.[35]; s: values for fruit considered; t: fitted from observations of Liu et al.[9]; u: Sun et al.[10]; v: Yang et al.[36]

Table S4. Parameterization of the Cell Model (exemplary parameters for plant cells).[20]

Parameters	Unit	Cytosol	Vacuole	Xylem	Phloem
Volume	m^3	10^{-4}	9×10^{-4}	2.33×10^{-5}	2.33×10^{-5}
Water content	kg/kg	0.943	0.943	1	1
Ion strength	mol/L	0.3	0.3	0.01	0.3
Lipid content	kg/kg	0.02	0.02	0	0
pH	-	7.4	5.0	5.5	8.0
Membrane potential to outside	V	-0.12 †	0.02 ‡	0.12 ‡	10^{-6} ‡

†: in root, outside is soil water; in the other plant compartments, outside is xylem apoplast; ‡: outside is cytosol

Table S5. Soil input data (generic assumptions).

Parameter	Symbol	Value	Unit
Leaching rate †	Q_l	0.27	L/d
Surface water runoff rate ‡	Q_r	0	L/d
Soil total porosity	n	0.45	L/L
Soil water content §	θ	0.45	L/L
Content of soil organic carbon	f_{OC}	0.015	g/g
Dry soil density	$\rho_{S,dry}$	1.58	kg/L
Soil depth	d	0.3	m
Soil pH	pH_S	7.6	-

Table S6: Physicochemical properties of the applied pesticides (Danish QSAR[37], unless noted differently).

Compound	CAS number	M_W (g/mol)	log K_{OW} (-)	log D at pH 7 (-) †	log D at pH 4 (-) †	K_{AW} (L/L)	pK_a acid (-)	pK_a base (-)	K_{HSA} (L/kg)
Imidacloprid	138261-41-3	255.66	0.38	0.38	0.38	6.75×10^{-14}	11.1	0.5	15.2
Spiromesifen	283594-90-1	370.48	5.25	5.25	5.25	9.55×10^{-06}	n.a.	n.a.	1785.9
Tetraniliprole	1229654-66-3	544.89	1.84	1.84	1.49	4.70×10^{-13}	n.a.	9.0	15.2 ‡
Thiacloprid	111988-49-9	252.72	0.98	0.98	-1.46	1.00×10^{-08}	n.a.	7.0	5647.4

M_W: molar mass, K_{OW}: octanol to water partition coefficient, D: apparent octanol to water partition coefficient, K_{AW}: air to water partition coefficient, pK_a: acid dissociation constant, K_{HSA}: partition coefficient to human serum albumin. †: Danish QSAR[37], obtained from ACD, ‡: value of imidacloprid used; n.a.: not applicable.

S5 Simulation results for individual experiments

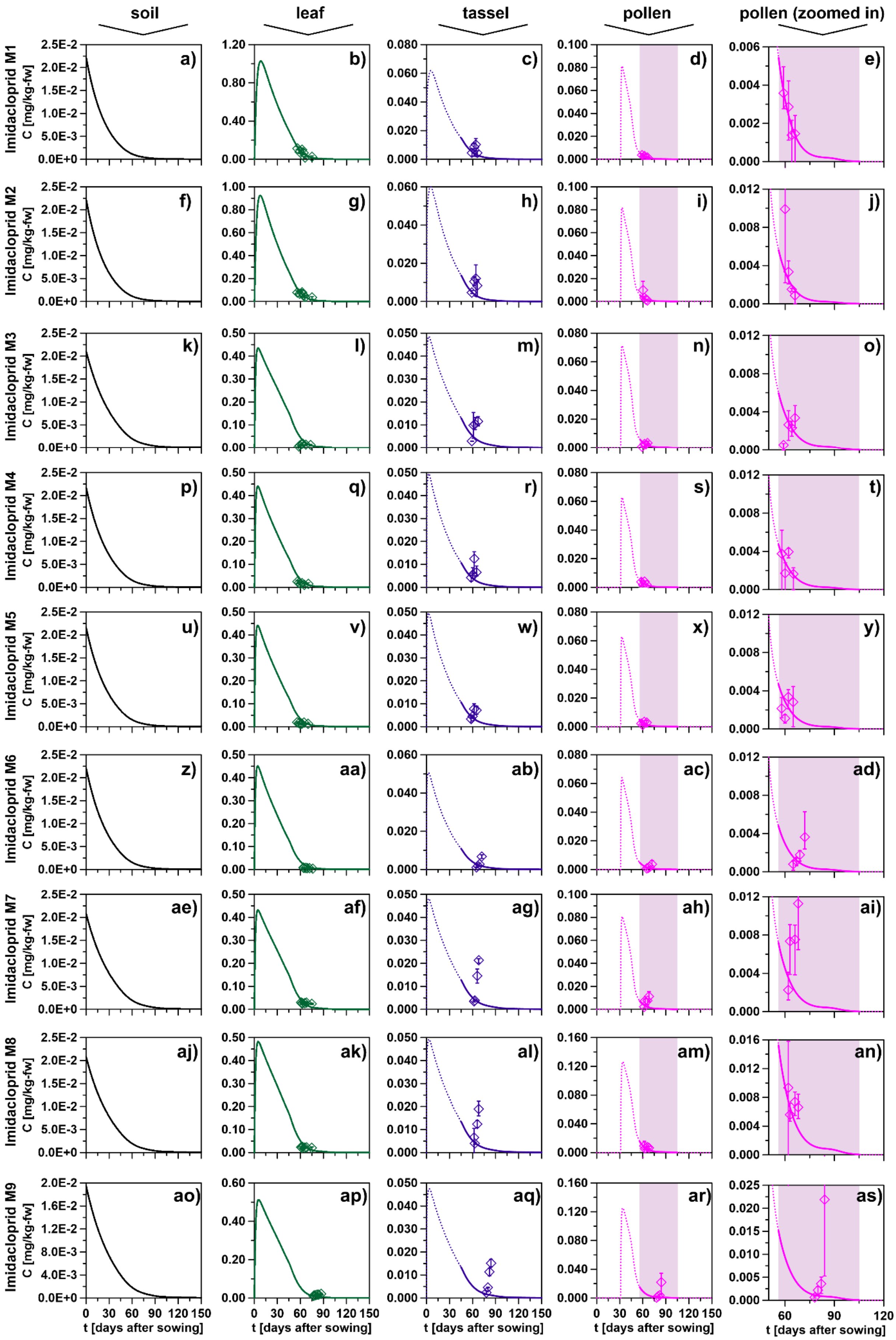

Figure S3: Modeling of nine field experiments with maize and **imidacloprid** (Miller et al.[1], seed treatment, cf. Table S1). Observed (symbols) vs. simulated (curves) concentrations in **soil** (a, f, k, p, u, z, ae, aj, ao), **leaf** (b, g, l, q, v, aa, af, ak, ap), **tassel** (c, h, m, r, w, ab, ag, al, aq) and **pollen** (d, e, i, j, n, o, s, t, x, y, ac, ad, ah, ai, am, an, ar, as). Observations: average; error bars indicate minimum and maximum (3-5 replicates). Fitted parameters and statistical curve fit evaluation in Table S7.

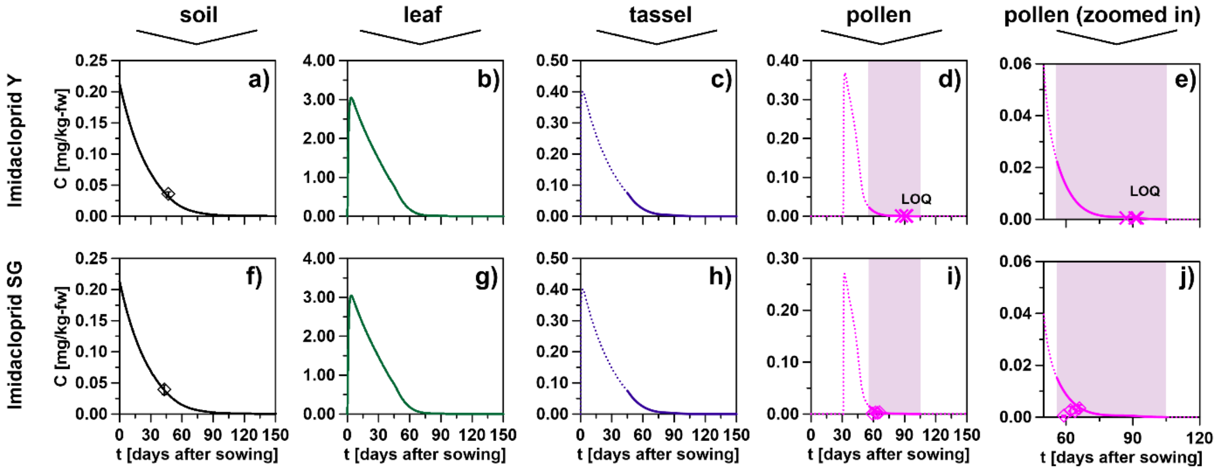

Figure S4: Modeling of field experiments with maize and **imidacloprid** (Y: Ythier et al.[2], SG: Schoening and Gerhardt[3]; no application (treatment in previous year), cf. Table S1). Observed (symbols) vs. simulated (curves) concentrations in soil (a, f), leaf (b, g), tassel (c, h) and pollen (d, e, i, j). Observations: average; error bars indicate minimum and maximum (3-5 replicates). Fitted parameters and statistical curve fit evaluation in Table S7. Experiment Imidacloprid Y, pollen: level of quantification (LOQ) shown; observed concentrations were below LOQ.

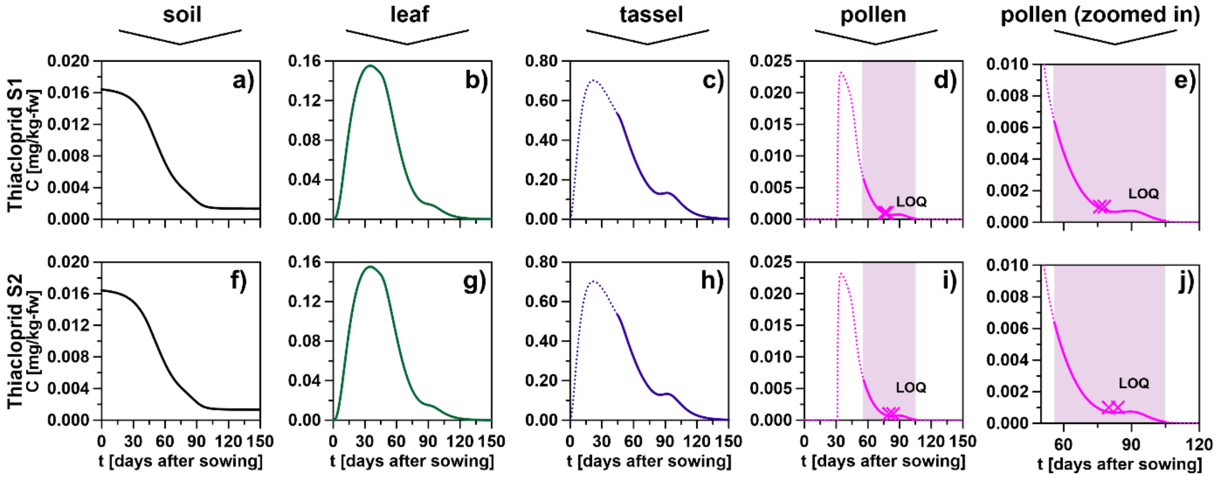

Figure S5: Modeling of two field experiments with maize and **thiacloprid** (Schoening[4], seed treatment, cf. Table S1). Simulated (curves) versus observed (symbols) concentrations in soil (a, f), leaf (b, g), tassel (c, h) and pollen (d, e, i, j). Observations, pollen: level of quantification (LOQ) shown; observed concentrations were below LOQ. Fitted parameters and statistical curve fit evaluation in Table S8.

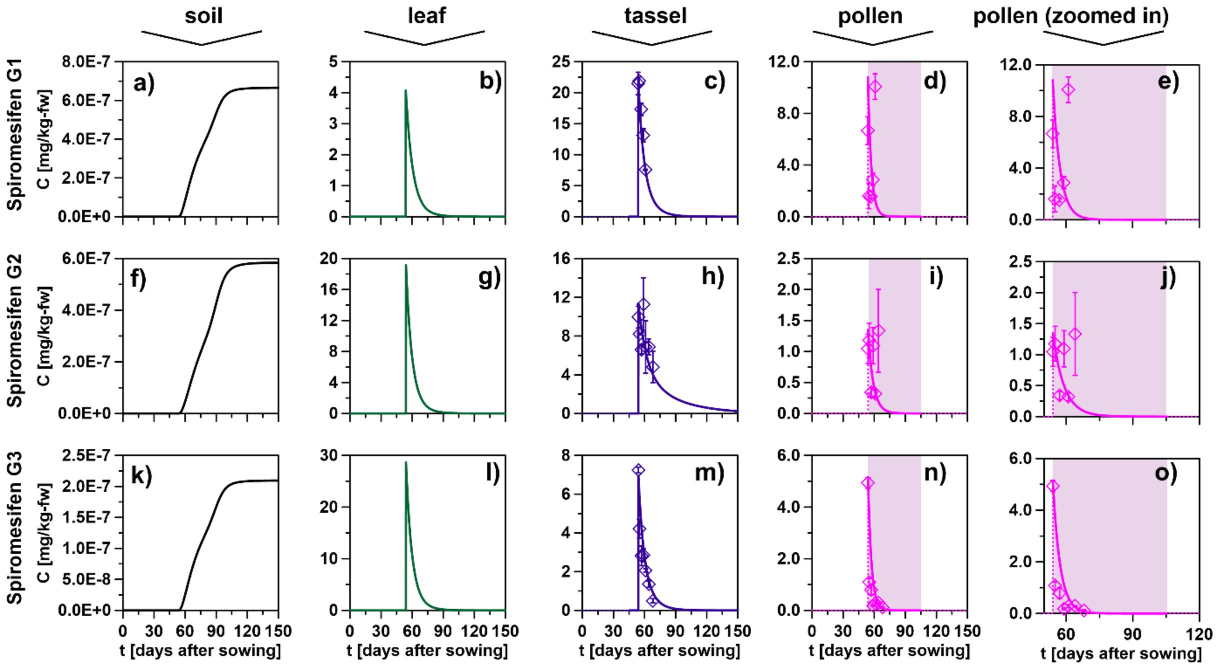

Figure S6: Modeling of three field experiments with maize and **spiromesifen** (Gould and Jerkins[7], foliar spray application, cf. Table S1). Observed (symbols) vs. simulated (curves) concentrations in soil (a, f, k), leaf (b, g, l), tassel (c, h, m) and pollen (d, e, i, j, n, o). Observations: average; error bars indicate minimum and maximum (3-5 replicates). Fitted parameters and statistical curve fit evaluation in Table S8.

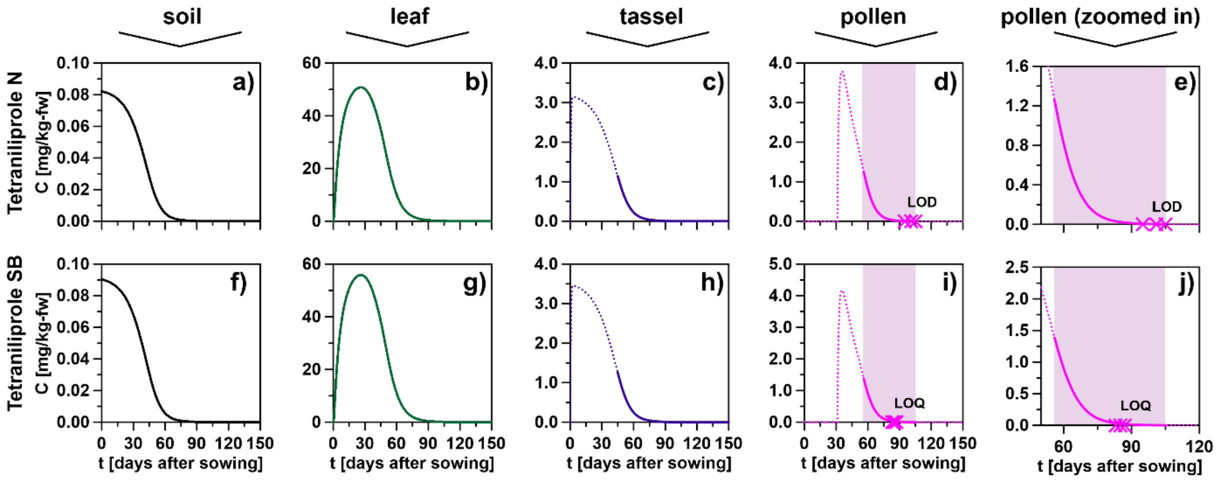

Figure S7: Modeling of two field experiments with maize and **tetraniliprole** (N: Noel et al.[5], SB: Striffler and Ballhaus[6]; soil incorporation and soil spray, respectively, cf. Table S1). Observed (symbols) vs. simulated (curves) concentrations in soil (a, f), leaf (b, g), tassel (c, h) and pollen (d, e, i, j). Observations, pollen: level of detection (LOD) and level of quantification (LOQ) shown; observed concentrations were below LOD and below LOQ, respectively. Fitted parameters and statistical curve fit evaluation in Table S8.

Table S7. Simulation of individual experiments with **imidacloprid** (IM) (cf. Figure S3 and 4). Total input of chemicals at application and fitted individual input to soil and plant, fitted loss rate constants used by the model for soil and plant, as well as empirical dissipation rate constants fitted to simulated concentrations together with dissipation half-lives. R^2: coefficient of determination, RSME: root mean square error, ME: mean error. With indices S for soil, St for stem, R for root, L for leaf, F for fruit, T for tassel, Fl for flower and Po for pollen.

**Compound**	**IM**	**IM**	**IM**	**IM**	**IM**	**IM**	**IM**	**IM**	**IM**	**IM**	**IM**
**Experiment**	**M1**	**M2**	**M3**	**M4**	**M5**	**M6**	**M7**	**M8**	**M9**	**SG**	**Y**
**Application**	**Seed †**	**Seed †**	**Seed †**	**Seed †**	**Seed †**	**Seed †**	**Seed †**	**Seed †**	**Seed †**	**None ‡**	**None ‡**
**Input (mg):**											
**Total**	13.5	13.5	12.8	13.2	13.2	13.5	12.7	12.7	11.9	n.r.	n.a.
**To soil**	13.5	13.5	12.8	13.2	13.2	13.5	12.7	12.7	11.9	35-130	35-130
**To stem**	0	0	0	0	0	0	0	0	0	0	0
**To leaf**	0	0	0	0	0	0	0	0	0	0	0
**To fruit**	0	0	0	0	0	0	0	0	0	0	0
**To flower**	0	0	0	0	0	0	0	0	0	0	0
**Model loss rate constants k (1/d):**											
**Soil (k_S)**	0.04	0.04	0.03	0.035	0.035	0.035	0.03	0.03	0.03	0-0.035	0-0.035
**Root (k_R)**	0	0	0	0	0	0	0	0	0	1	1
**Stem (k_{St}**	0.1	0.05	0.05	0.05	0.05	0.05	0.05	0.05	0.05	1	1
**Leaf (k_L)**	0.25	0.3	0.8	0.8	0.8	0.8	0.7	0.7	0.6	1	1
**Fruit (k_F)**	0	0	0	0	0	0	0	0	0	0	0
**Flower (k_{Fl})**	2	1.8	1.8	1.8	1.8	1.8	1.5	0.8	0.8	3.5	2.4
**Dissipation rate constants k_{diss} (1/d) fitted to simulated concentrations:**											
**Stem & tassel**	0.05	0.05	0.05	0.05	0.05	0.05	0.05	0.05	0.05	0.065	0.065
**Leaf**	0.07	0.075	0.075	0.08	0.085	0.08	0.075	0.078	0.075	0.09	0.09
**Pollen & flower**	0.12	0.12	0.13	0.12	0.12	0.12	0.11	0.10	0.10	0.14	0.14
**Half-lives $t_{1/2}$ (d) (with $t_{1/2}$ = ln(2) / k_{diss}):**											
**Stem & tassel**	13.9	13.9	13.9	13.9	13.9	13.9	13.9	13.9	13.9	10.7	10.7
**Leaf**	9.9	9.2	9.2	8.7	8.2	8.7	9.2	8.9	9.2	7.7	7.7
**Pollen & flower**	5.8	5.8	5.3	5.8	5.8	5.8	6.3	6.9	6.9	5.0	5.0
**Statistical curve fit evaluation (measured vs. modeled concentrations):**											
**R^2_S[-]**	n.a.	n.a.	n.a.	n.a.	n.a.	n.a.	n.a.	n.a.	n.a.	n.a.	n.a.
**ME_S [mg/kg]**	n.a.	n.a.	n.a.	n.a.	n.a.	n.a.	n.a.	n.a.	n.a.	-0.0012	-0.0048
**$RMSE_S$ [mg/kg]**	n.a.	n.a.	n.a.	n.a.	n.a.	n.a.	n.a.	n.a.	n.a.	0.0012	0.0048
**R^2_L [-]**	0.720	0.697	0.288	0.618	0.154	0.832	0.232	0.303	0.975	n.a.	n.a.
**ME_L [mg/kg]**	0.0057	0.0023	0.0227	0.0139	0.0163	0.0082	-0.0004	0.0086	-0.0083	n.a.	n.a.
**$RMSE_L$ [mg/kg]**	0.0206	0.0190	0.0289	0.0174	0.0207	0.0095	0.0099	0.0144	0.0095	n.a.	n.a.
**R^2_T [-]**	0.057	0.417	0.718	0.219	0.773	0.853	0.972	0.869	0.956	n.a.	n.a.
**ME_T [mg/kg]**	-0.0030	-0.0052	-0.0042	-0.0036	-0.0020	-0.0008	-0.0070	-0.0066	-0.0069	n.a.	n.a.
**$RMSE_T$ [mg/kg]**	0.0042	0.0062	0.0059	0.0050	0.0032	0.0027	0.0107	0.0091	0.0087	n.a.	n.a.
**R^2_{Po} [-]**	0.886	0.893	0.868	0.199	0.290	0.840	0.787	0.153	0.534	0.872	n.a.
**ME_{Po} [mg/kg]**	0.0000	-0.0016	0.0006	-0.0002	0.0002	-0.0007	-0.0045	-0.0017	-0.0061	0.0041	0.0001
**$RMSE_{Po}$ [mg/kg]**	0.0003	0.0033	0.0020	0.0010	0.0015	0.0016	0.0059	0.0024	0.0106	0.0054	0.0002

IM: imidacloprid; M1-M9: Miller et al. [1] (nine experiments), SG: Schoening and Gerhardt [3], Y: Ythier et al. [2]; †: seed treatment, ‡: treatment in previous year (thus residues in soil); n.r.: not reported; n.a.: not applicable.

Table S8. Simulation of individual experiments with **thiacloprid** (TH), **spiromesifen** (SP) and **tetraniliprole** (TE) (cf. Figure S4, S5 and 6). Total input of chemicals at application and fitted individual input to soil and plant, fitted fitted loss rate constants used by the model for soil and plant, as well as empirical dissipation rate constants fitted to simulated concentrations together with dissipation half-lives. R^2: coefficient of determination, RSME: root mean square error, ME: mean error. With indices S for soil, St for stem, R for root, L for leaf, F for fruit, T for tassel, Fl for flower and Po for pollen.

**Compound**<br>**Experiment**<br>**Application**	**TH**<br>**S1**<br>**Seed †**	**TH**<br>**S2**<br>**Seed †**	**SP**<br>**G1**<br>**Foliar ‡**	**SP**<br>**G2**<br>**Foliar ‡**	**SP**<br>**G3**<br>**Foliar ‡**	**TE**<br>**N**<br>**Soil in.§**	**TE**<br>**SB**<br>**Soil sp.¶**
**Input (mg):**							
**Total**	10	10	27.7	29.3	28	15	15.3
**To soil**	10	10	0	0	0	50	55
**To stem**	0	0	25	18	11	0	0
**To leaf**	0	0	2.4	11.3	16.9	0	0
**To fruit**	0	0	0	0	0	0	0
**To flower**	0	0	0.32	0.04	0.15	0	0
**Model loss rate constants k (1/d):**							
**Soil (k_S)**	0	0	0	0	0	0	0
**Root (k_R)**	0	0	0	0	0	0	0
**Stem (k_{St}**	0.1	0.1	0.1	0.03	0.1	0.1	0.1
**Leaf (k_L)**	0.1	0.1	0.1	0.1	0.1	0.1	0.1
**Fruit (k_F)**	0	0	0	0	0	0	0
**Flower (k_{Fl})**	0.8	0.8	0.3	0.2	0.3	0.2	0.2
**Dissipation rate constants k_{diss} (1/d) fitted to simulated concentrations:**							
**Stem & tassel**	0.045	0.05	0.14	0.06	0.13	0.12	0.11
**Leaf**	0.06	0.065	0.095	0.06	0.1	0.12	0.1
**Pollen & flower**	0.12	0.13	0.3	0.2	0.3	0.12	0.11
**Half-lives $t_{1/2}$ (d) (with $t_{1/2}$ = ln(2) / k_{diss}):**							
**Stem & tassel**	15.4	13.9	5.0	11.6	5.3	5.8	6.3
**Leaf**	11.6	10.7	7.3	11.6	6.9	5.8	6.9
**Pollen & flower**	5.8	5.3	2.3	3.5	2.3	5.8	6.3
**Statistical curve fit evaluation (measured vs. modeled concentrations):**							
**R^2_S [-]**	n.a.	n.a.	n.a.	n.a.	n.a.	n.a.	n.a.
**ME_S [mg/kg]**	n.a.	n.a.	n.a.	n.a.	n.a.	n.a.	-0.0026
**$RMSE_S$ [mg/kg]**	n.a.	n.a.	n.a.	n.a.	n.a.	n.a.	0.0098
**R^2_T [-]**	n.a.	n.a.	0.898	0.360	0.896	n.a.	n.a.
**ME_T [mg/kg]**	n.a.	n.a.	-1.7834	-0.1561	0.6062	n.a.	n.a.
**$RMSE_T$ [mg/kg]**	n.a.	n.a.	2.4950	2.0703	0.9064	n.a.	n.a.
**R^2_{Po} [-]**	n.a.	n.a.	0.032	0.018	0.741	n.a.	n.a.
**ME_{Po} [mg/kg]**	-0.0002	-0.0003	0.8133	-0.1809	0.7448	0.0019	0.0227
**$RMSE_{Po}$ [mg/kg]**	0.0002	0.0003	5.3378	0.5661	1.1686	0.0026	0.0232

S1-S2: Schoening [4] (two experiments), G1-G3: Gould and Jerkins [7] (three experiments), N: Noel et al. [5], SB: Striffler and Ballhaus [6]; †: seed treatment, ‡: foliar application (1 spray), §: soil incorporation, ¶: soil spray; n.r.: not reported; n.a.: not applicable.

REFERENCES

(1) Miller, A.; Bowers, I.; Dyer, D.; Jerkins, E. *Determination of the residues of imidacloprid and its metabolites 5-hydroxy imidacloprid and imidacloprid olefin*; Bayer AG unpublished report M-500863-01-2, 2014.

(2) Ythier, E. *Determination of the residues of imidacloprid and its metabolites imidacloprid-5-hydroxy and imidacloprid-olefin in bee relevant matrices collected in a succeeding crop scenario with natural aged residues of imidacloprid*; Bayer AG unpublished report M-502448-01-1, 2014.

(3) Schoening, R.; Gerhardt, N. *Determination of the residues of imidacloprid and its metabolites imidacloprid-5-hydroxy and imidacloprid-olefin in bee relevant matrices collected in a succeeding crop scenario with natural aged residues of imidacloprid*; Bayer AG unpublished report M-502498-01-1, 2014.

(4) Schoening, R. *Determination of residue levels of thiacloprid and its metabolite KKO 2254 in pollen, harvested from maize plants, grown from Thiacloprid FS 400 dressed seeds (nominally 1.00 mg thiacloprid/seed) in Germany*; Bayer AG unpublished report M-363263-01-1, 2010.

(5) Noel, E. *Determination of the residues of tetraniliprole and its metabolite tetraniliprole-N-methyl-quinazolinone in bee relevant matrices collected from succeeding crops following applications of tetraniliprole SC 200 G (200 g/L) via soil incorporation*; Bayer AG unpublished report M-650694-01-1, 2019.

(6) Striffler, B.; Ballhaus, F. *Residues of BCS-CL73507 in guttation fluid, nectar and pollen of flowering rotational crops in Western Germany*; Bayer AG unpublished report M-620354-01-2, 2018.

(7) Gould, T.; Jerkins, E. *Determination of the residues of spiromesifen and its metabolite spiromesifen enol in bee relevant matrices collected from sweet corn following foliar application of Oberon 240 SC*; Bayer AG unpublished report M-586997-01-1, 2017.

(8) Koca, Y. O.; Erekul, O. Changes of Dry Matter, Biomass and Relative Growth Rate with Different Phenological Stages of Corn. *Agriculture and Agricultural Science Procedia* **2016**, *10*, 67–75. DOI: https://doi.org/10.1016/j.aaspro.2016.09.015.

(9) Liu, P.; Yin, B.; Gu, L.; Zhang, S.; Ren, J.; Wang, Y.; Duan, W.; Zhen, W. Heat stress affects tassel development and reduces the kernel number of summer maize. *Frontiers in Plant Science* **2023**, *14*, 1–13, Original Research. DOI: 10.3389/fpls.2023.1186921.

(10) Sun, J.; Wang, H.; Ren, H.; Zhao, B.; Zhang, J.; Ren, B.; Liu, P. Maize (Zea mays L.) responses to heat stress: Mechanisms that disrupt the development and hormone balance of tassels and pollen. *Journal of Agronomy and Crop Science* **2023**, *209* (4), 502–516. DOI: https://doi.org/10.1111/jac.12644.

(11) Rein, A.; Trapp, S.; Fantke, P.; Yalçın, M.; Turgut, N.; Ahat, C.; Camcı, E.; Turgut, C. Uptake and translocation of pesticides in pepper and tomato plants. *Pest Management Science* **2025**, *81* (3), 1562–1570. DOI: 10.1002/ps.8556.

(12) Gredelj, A.; Polesel, F.; Trapp, S. Model-based analysis of the uptake of perfluoroalkyl acids (PFAAs) from soil into plants. *Chemosphere* **2020**, *244*, 125534. DOI: 10.1016/j.chemosphere.2019.125534 From NLM Medline.

(13) Lamshoeft, M.; Gao, Z.; Resseler, H.; Schriever, C.; Sur, R.; Sweeney, P.; Webb, S.; Zillgens, B.; Reitz, M. U. Evaluation of a novel test design to determine uptake of chemicals by plant roots. *Sci Total Environ* **2018**, *613-614*, 10–19. DOI: 10.1016/j.scitotenv.2017.08.314 From NLM Medline.

(14) Trapp, S.; Shi, J.; Zeng, L. Generic Model for Plant Uptake of Ionizable Pharmaceuticals and Personal Care Products. *Environmental Toxicology and Chemistry* **2023**, *42* (4), 793–804. DOI: https://doi.org/10.1002/etc.5582.

(15) Trapp, S. Fruit Tree model for uptake of organic compounds from soil and air. *SAR QSAR Environ Res* **2007**, *18* (3-4), 367–387. DOI: 10.1080/10629360701303693 From NLM.

(16) Rein, A.; Legind, C. N.; Trapp, S. New concepts for dynamic plant uptake models. *SAR QSAR Environ Res* **2011**, *22* (1-2), 191–215. DOI: 10.1080/1062936X.2010.548829 From NLM Medline.

(17) Trapp, S.; Matthies, M. *Chemodynamics and Environmental Modeling: An Introduction,* Springer: Berlin, Heidelberg, 1998. DOI: 10.1007/978-3-642-80429-8.

(18) EC European Commission. *Technical Guidance Document in Support of Commission Directive 93/67/EEC on Risk Assessment for New Notified Substances and Commission Regulation (EC) No 1488/94 on Risk Assessment for Existing Substances*; Luxemburg, 1996.

(19) Franco, A.; Trapp, S. Estimation of the soil–water partition coefficient normalized to organic carbon for ionizable organic chemicals. *Environmental Toxicology and Chemistry* **2008**, *27* (10), 1995–2004. DOI: https://doi.org/10.1897/07-583.1 (accessed 2024/03/14).

(20) Trapp, S. Modelling uptake into roots and subsequent translocation of neutral and ionisable organic compounds. *Pest Management Science* **2000**, *56* (9), 767–778. DOI: https://doi.org/10.1002/1526-4998(200009)56:9<767::AID-PS198>3.0.CO;2-Q (accessed 2024/03/14).

(21) Trapp, S. Plant uptake and transport models for neutral and ionic chemicals. *Environmental Science and Pollution Research* **2004**, *11* (1), 33–39. DOI: 10.1065/espr2003.08.169.

(22) Trapp, S.; Horobin, R. W. A predictive model for the selective accumulation of chemicals in tumor cells. *Eur Biophys J* **2005**, *34* (7), 959–966. DOI: 10.1007/s00249-005-0472-1 From NLM.

(23) Legind, C. N.; Rein, A.; Serre, J.; Brochier, V.; Haudin, C. S.; Cambier, P.; Houot, S.; Trapp, S. Simultaneous simulations of uptake in plants and leaching to groundwater of cadmium and lead for arable land amended with compost or farmyard manure. *PLoS One* **2012**, *7* (10), e47002. DOI: 10.1371/journal.pone.0047002 From NLM.

(24) Trapp, S. Dynamic root uptake model for neutral lipophilic organics. *Environ Toxicol Chem* **2002**, *21* (1), 203–206. From NLM.

(25) Song, J.; Lu, D.; Niu, Y.; Sun, H.; Zhang, P.; Dong, W.; Li, Y.; Zhang, Y.; Lu, L.; Men, Q.; et al. Label-free quantitative proteomics of maize roots from different root zones provides insight into proteins associated with enhance water uptake. *BMC Genomics* **2022**, *23* (1), 184. DOI: 10.1186/s12864-022-08394-y.

(26) FeedTables. *Maize composition*. 2025. https://feedtables.com/content/maize (accessed 4 November 2025).

(27) Shu, M.; Zhou, L.; Chen, H.; Wang, X.; Meng, L.; Ma, Y. Estimation of amino acid contents in maize leaves based on hyperspectral imaging. *Frontiers in Plant Science* **2022**, *13*, 885794. DOI: 10.3389/fpls.2022.885794.

(28) Yu-Kui, R.; Yun-Feng, P.; Zheng-Rui, W.; Jian-Bo, S. Stem perimeter, height and biomass of maize (Zea mays L.) grown under different N fertilization regimes in Beijing, China. *International Journal of Plant Production* **2009**, *3* (2), 85–90.

(29) Zhou, H.; Zhou, G.; He, Q.; Zhou, L.; Ji, Y.; Zhou, M. Environmental explanation of maize specific leaf area under varying water stress regimes. *Environmental and Experimental Botany* **2020**, *171*, 103932. DOI: https://doi.org/10.1016/j.envexpbot.2019.103932.

(30) Danalatos, N.; Kosmas, C.; Driessen, P.; Yassoglou, N. The change in the specific leaf area of maize grown under Mediterranean conditions. *Agronomie* **1994**, *14* (7), 433–443.

(31) Barrera-Arellano, D.; Badan-Ribeiro, A. P.; Serna-Saldivar, S. O. Chapter 21 - Corn Oil: Composition, Processing, and Utilization. In *Corn (Third Edition)*, Serna-Saldivar, S. O. Ed.; AACC International Press, 2019; pp 593–613.

(32) FAO Food and Agriculture Organization. Maize in human nutrition. In *Food and Nutrition Series 25*, FAO: Rome, Italy, 1992.

(33) Iqbal, M. A.; Ahmad, Z.; Maqsood, Q.; Afzal, S.; Ahmad, M. M. Optimizing nitrogen level to improve growth and grain yield of spring planted irrigated maize (Zea mays L.). *J. Adv. Bot. Zool. J* **2015**, *2* (3), 1–4. DOI: 10.15297/JABZ.V2I3.02.

(34) Sangamithra, A.; Swamy, G. J.; Sorna Prema, R.; Nandini, K.; Kannan, K.; Sasikala, S.; Suganya, P. Moisture dependent physical properties of maize kernels. *International Food Research Journal* **2016**, *23* (1), 109–115.

(35) Fonseca, A. E.; Westgate, M. E.; Grass, L.; Dornbos Jr, D. L. Tassel morphology as an indicator of potential pollen production in maize. *Crop Management* **2003**, *2* (1), 1–15. DOI: 10.1094/CM-2003-0804-01-RS.

(36) Yang, K.; Wu, D.; Ye, X.; Liu, D.; Chen, J.; Sun, P. Characterization of chemical composition of bee pollen in China. *Journal of Agricultural and Food Chemistry* **2013**, *61* (3), 708–718. DOI: 10.1021/jf304056b.

(37) Danish QSAR. *Danish QSAR database*. 2025. http://qsar.food.dtu.dk (accessed 15 July 2025).
